# m6A/m1A/m5C-Associated Methylation Alterations and Immune Profile in MDD

**DOI:** 10.1007/s12035-024-04042-6

**Published:** 2024-03-08

**Authors:** Xin Ren, Zhuxiao Feng, Xiaodong Ma, Lijuan Huo, Huiying Zhou, Ayu Bai, Shujie Feng, Ying Zhou, Xuchu Weng, Changhe Fan

**Affiliations:** 1 Key Laboratory of Brain, Cognition and Education Sciences, Ministry of Education, 55 Zhongshan Avenue West, Tianhe District, Guangzhou, 510631 China; 2 Institute for Brain Research and Rehabilitation, South China Normal University, Guangzhou, 510631 China; 3 Department of Psychiatry, Affiliated Guangdong Second Provincial General Hospital of Jinan University, Guangzhou, 510317 China; 4 Department of Rehabilitation Medicine, Affiliated Guangdong Second Provincial General Hospital of Jinan University, Guangzhou, 510317 China

**Keywords:** RNA modification, Diagnostic, Major depressive disorder, Immune, Gene cluster analysis, NUDT21, IGF2BP1

## Abstract

Major depressive disorder (MDD) is a prevalent psychiatric condition often accompanied by severe impairments in cognitive and functional capacities. This research was conducted to identify RNA modification-related gene signatures and associated functional pathways in MDD. Differentially expressed RNA modification-related genes in MDD were first identified. And a random forest model was developed and distinct RNA modification patterns were discerned based on signature genes. Then, comprehensive analyses of RNA modification-associated genes in MDD were performed, including functional analyses and immune cell infiltration. The study identified 29 differentially expressed RNA modification-related genes in MDD and two distinct RNA modification patterns. TRMT112, MBD3, NUDT21, and IGF2BP1 of the risk signature were detected. Functional analyses confirmed the involvement of RNA modification in pathways like phosphatidylinositol 3-kinase signaling and nucleotide oligomerization domain (NOD)-like receptor signaling in MDD. NUDT21 displayed a strong positive correlation with type 2 T helper cells, while IGF2BP1 negatively correlated with activated CD8 T cells, central memory CD4 T cells, and natural killer T cells. In summary, further research into the roles of NUDT21 and IGF2BP1 would be valuable for understanding MDD prognosis. The identified RNA modification-related gene signatures and pathways provide insights into MDD molecular etiology and potential diagnostic biomarkers.

**Supplementary Information:**

The online version contains supplementary material available at 10.1007/s12035-024-04042-6.

## Introduction

Major depressive disorder (MDD) is widely encountered in psychiatric practice, exhibiting a formidable global 12-month incidence rate of 4.4% [1]. This multifaceted syndrome is characterized by diverse symptoms, including a pervasive sense of melancholy, a pronounced reduction in pleasure-seeking tendencies, fluctuations in weight and sleep patterns, heightened fatigue, self-deprecatory cognition, and notable cognitive challenges, particularly in areas of focus and decision-making. Additionally, it is associated with an increased predisposition toward morbid ideation or suicidal thoughts [2]. Despite its prevalence, effective treatment and prevention strategies for MDD remain elusive. Contemporary diagnostic approaches largely rely on subjective symptomatology reported by patients and clinician assessments, lacking concrete biological markers [3]. Such an approach not only complicates differential diagnosis but also increases the risk of oversight. Given these challenges, there is a compelling need to elucidate the molecular underpinnings of MDD and identify groundbreaking biomarkers to enhance diagnostic precision and therapeutic outcomes.

Recent research emphasizes the pivotal role of epigenetic mechanisms, influenced by environmental and developmental cues, in modulating gene activity. Numerous epigenetic aberrations have been identified in MDD, notably including DNA methylation, RNA modifications, chromatin restructuring, the involvement of noncoding RNAs, and histone adjustments [4]. These alterations often target genes crucial for the formation, operation, and adaptability of neuronal networks within the central nervous system (CNS) [5, 6]. Despite their significance, RNA modifications in the epigenetic landscape of MDD have received relatively little attention. These modifications, which play a critical role in influencing RNA–protein interactions, underlie essential post-transcriptional gene expression regulation processes [7]. Notably, the most frequently observed RNA modifications include N6-adenylate methylation (m6A), N1-adenylate methylation (m1A), and cytosine hydroxylation (m5C) [8]. Particularly, m6A levels have been observed to increase in the murine cerebral cortex following stress, correlating with transcriptional variations in neuronal genes. These stress-induced m6A alterations significantly overlap with genomic regions associated with neuropsychiatric disorders [9]. In MDD patients, the regulatory dynamics of m6A are found to be compromised after exposure to glucocorticoids. The orchestration of the m6A epitranscriptome is principally mediated by the methyltransferase methyltransferase-like protein 3 (METTL3) and the demethylase fat mass and obesity-associated protein (FTO), both playing a central role in modulating fear-associated transcriptional responses [10]. Unlike m6A, m1A methylates adenylate at the N1 position. Previous studies have revealed that dysregulation of m1A is closely associated with psychiatric disorders [11]. The m5C RNA modification plays an instrumental role in modulating mRNA stability, expression, and translational mechanisms. Specifically, m5C’s influence on tRNAs, particularly tRNA^Gly^, adds another layer of epitranscriptomic regulation significant for the mature brain’s neurobiological functions and behavioral tendencies [12]. It is worth noting that there is a gap in the bioinformatics domain concerning m6A/m5C/m1A in the context of MDD. Therefore, a comprehensive study of genes related to m6A/m5C/m1A in MDD is crucial for developing a refined prognostic framework and identifying potential diagnostic markers.

In our investigation, RNA sequencing data specific to MDD patients were obtained from GEO repositories, with the aim of exploring the implications of genes linked to m6A/m5C/m1A in MDD. Following the development of both forest and nomogram models, validation was performed using additional Gene Expression Omnibus (GEO) datasets and clinical specimens from individuals diagnosed with MDD. Utilizing the Uniform Manifold Approximation and Projection (UMAP) algorithm, distinct RNA modification profiles anchored on m6A/m5C/m1A-definitive genes were discerned. Furthermore, through functional enrichment analysis, dichotomous patterns of RNA modification-associated genes within MDD were revealed. Simultaneously, a patient and public involvement (PPI) framework was established, and the associative dynamics between transcription factors (TFs) and central genes were delineated, leveraging both the Search Tool for the Retrieval of Interacting Genes (STRING) and miRNet databases. In conclusion, we conducted a comprehensive analysis, emphasizing the interplay between RNA modification-associated genetic signatures and immune cell integration.

Given the urgent need to unravel the intricate etiology and molecular underpinnings of MDD, our findings provide insights into the molecular pathways and prognostic indicators associated with RNA modifications in MDD.

## Materials and Methods

### Data Download

**Fig. 1:**
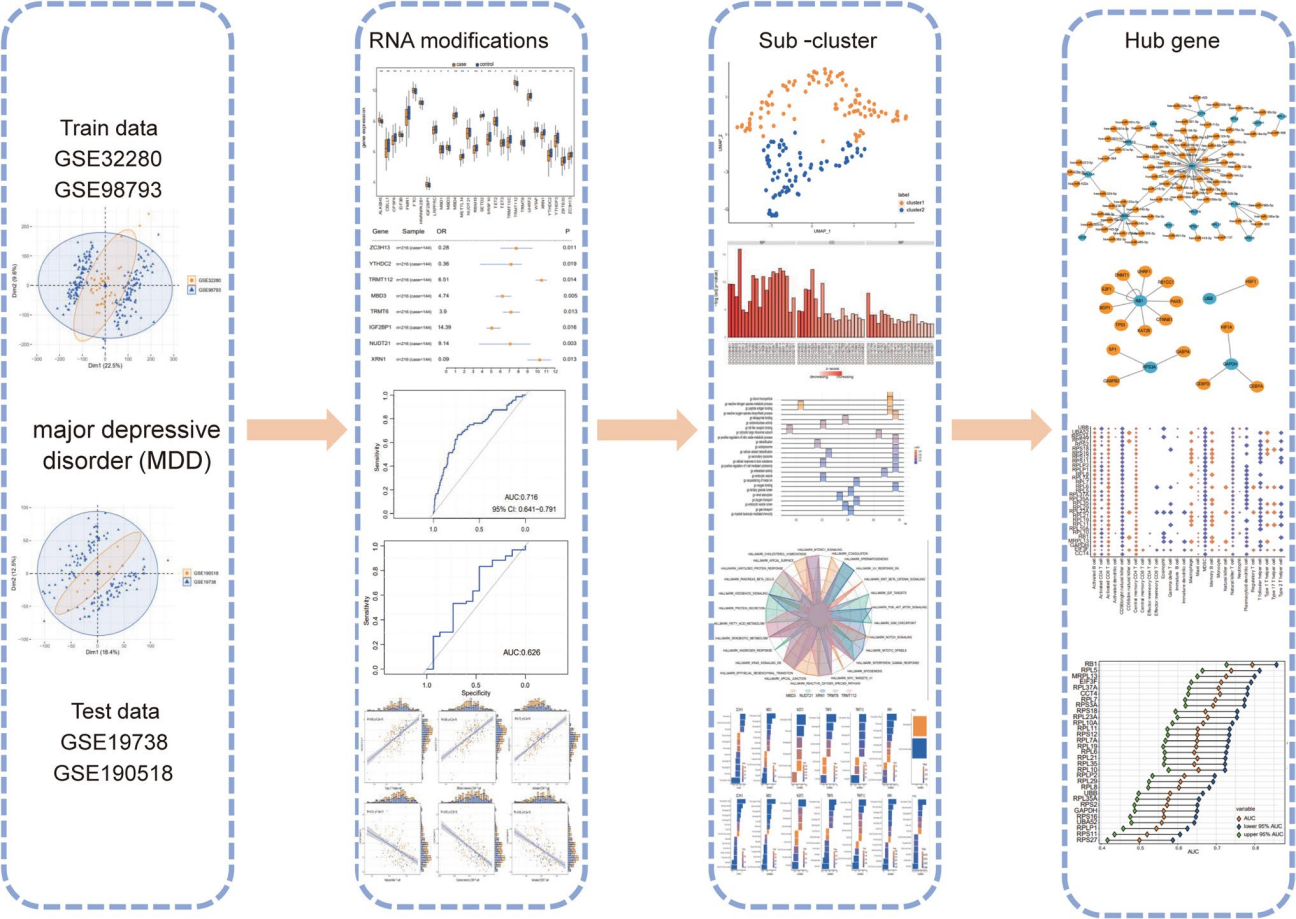
Flowchart of the study design. DEGs, differentially expressed genes; GO, Gene Ontology; KEGG, Kyoto Encyclopedia of Genes and Genomes; PPI, protein–protein interaction; GSEA, Gene Set Enrichment Analysis; GSEA, gene set enrichment analysis; ssGSEA, single-sample gene set enrichment analysis

As the analysis flow diagram (Fig. 1), we embarked on a comprehensive evaluation. With the aim of identifying genes expressed in MDD and matched controls, the GEO repository—one of the world’s most extensive collections of gene chips—was utilized to acquire and integrate four gene expression profiles (GSE32280 [13], GSE98793 [14], GSE19738 [15], and GSE190518 [16]). Homo sapiens were selected as the subject, and the platforms used were GPL570 and GPL6848. Specifically, GSE32280 contained 16 depression samples and 8 control samples; GSE98793 contained 128 depression samples and 64 control samples; GSE19738 comprised 38 depression samples and 37 control samples; GSE190518 included 38 depression samples and 37 control samples. Subsequently, GSE32280 and GSE98793 datasets were combined as the training set for the diagnostic model, and GSE19738 and GSE190518 were combined as the validation set for the diagnostic model. Batch effects were normalized and removed using the R sva package [17]. Simultaneously, RNA modification-related genes were extracted from the literature [18], encompassing m1A-, m5C-, and m6A-related genes (Supplementary Table 1). Box plots (Table 1) were employed to visualize the expression distribution.

**Table 1:** Summary of the GEO dataset information

Data set classification	GSE	GPL	Species	Organization source	Sample number of MDD	Sample number of control	Reference
Training set	GSE32280	GPL570	Homo sapiens	Peripheral blood	16	8	PMID: 22,348,066
	GSE98793	GPL570	Homo sapiens	Whole blood	128	64	PMID: 28,688,579
Test set	GSE19738	GPL6848	Homo sapiens	Whole blood	38	37	PMID: 20,471,630
	GSE190518	GPL20301	Homo sapiens	Cubital vein Peripheral whole blood	4	4	PMID: 35,431,783

### Identification of RNA Modification-related Differentially Expressed Genes

To examine the impact of RNA modification-associated gene expression magnitudes on MDD, the R package “limma” (version 3.58.1) was utilized for conducting a comparative gene analysis between MDD specimens and their standard counterparts within the combined dataset [19]. Criteria for identifying differentially expressed genes (DEGs) included an absolute fold change (FC) value exceeding 1.2 and a significance level (*P*) below 0.05. DEGs with FC greater than 1.2 and *P* less than 0.05 were categorized as upregulated, while those with FC less than − 1.2 and *P* below 0.05 were classified as downregulated. The identified set of DEGs was subsequently compared with RNA modification-associated genes to derive a set of differentially expressed RNA-modified genes (DERMG). Visual representation of DERMG outcomes was achieved through a volcanic plot.

### Development of the Forest and Nomogram Frameworks

The forest model was employed to select candidate signature genes from the DERMGs and predict the onset of MDD. The signature genes were incorporated into the model, and the risk score formula was formulated as follows:

$$\mathrm{Risk\;score}=\sum\mathrm{Coefficient}\;\left(\text{genei}\right)\;\ast\;\mathrm{mRNA}\;\mathrm{Expression}\;\left(\text{genei}\right)$$

A nomogram framework was constructed using the selected signature genes to predict the occurrence of MDD. Subsequently, an independent validation set was utilized to verify the accuracy of the model.

### Functional Enrichment Analysis (FEA) of DERMGs in MDD

The Gene Ontology (GO) framework continues to be a crucial tool in FEA for conducting comprehensive investigations, covering dimensions such as biological processes (BP), cellular components (CC), and molecular functions (MF) [20]. Kyoto Encyclopedia of Genes and Genomes (KEGG) is widely recognized as a repository that provides detailed information on biological pathways, genomic data, therapeutic agents, and associated disorders [21]. To enable GO annotations and KEGG enrichment assessments of DERMGs, the Cluster Profiler toolkit in R (version 4.10.0) was employed [22, 23]. A false discovery rate (FDR) value below 0.05 was considered indicative of statistical significance.

### Appraisal and Association Study of Immune Cell Penetration in MDD

The immune milieu, a complex assembly, is predominantly constituted by immune cells, inflammatory agents, fibroblasts, a spectrum of chemokines and cytokines, and the extracellular matrix. A profound understanding of the extent to which tissues are infiltrated by immune cells is of paramount importance in both disease research and prognostic prediction. Single-sample gene set enrichment analysis (ssGSEA), an advanced adaptation of the GSEA methodology, provides insights into this aspect. Additionally, Cell-type Identification by Estimating Relative Subsets of RNA Transcripts (CIBERSORT), employing the linear support vector regression paradigm, deciphers the transcriptional signatures of various immune cell subtypes. This algorithm facilitates the quantification of immune cell prevalence within tissues through RNA-Seq data analysis [24]. Using the CIBERSORT algorithm in the R environment, the relative abundance of 22 distinct immune cells was determined in both high-risk and low-risk sample sets. Subsequently, boxplot representations were used to visualize these immune cell distributions in both pathological and control specimens [25]. The Wilcoxon rank-sum test was employed to detect disparities in immune cell distribution between the diseased and healthy cohorts, with a significance threshold of *P* < 0.05.

The ESTIMATE methodology, designed to assess immune responsiveness (the degree of immune cell infiltration) within tumor specimens based on transcriptional data, offers insights into the concentration of stromal and immune-specific gene signatures [26].

To elucidate the complex interplay of RNA modification patterns in patients, the ssGSEA methodology was employed, enabling the quantification of 28 distinct immune cell types [24]. Furthermore, by leveraging the CIBERSORT algorithm within the R platform, the relative concentrations of 22 specific immune cells were discerned in the patient cohorts [25]. To delineate the relationship between depression-associated key genes and diverse immune signatures, the R corrplot package (version 1.29) was utilized to contextualize the findings from our immune infiltration assessments [27]. Subsequent analyses utilized the “estimate” package within R (version 1.0.13) to compare immune scoring across patient groups defined by their RNA modification profiles [26]. This was complemented by correlation studies examining the connection between central gene transcriptional levels and ESTIMATE values.

### Molecular Subtypes of MDD

The advanced dimensionality minimization technique, UMAP, has the capability to separate or streamline a cohort of patients into distinct clusters based on specific attributes. Utilizing the UMAP package (version 0.2.10.0) within R [28], varied RNA modification patterns rooted in characteristic genes were identified. These characteristic genes were designated as central RNA modulatory genes associated with MDD. These genes were defined as key RNA modification genes related to MDD.

### Analysis of Biological Traits Across Patients Exhibiting Distinct RNA Modification Profiles

The R package cluster was utilized for GO functional annotation and KEGG pathway analysis of DEGs among the MDD cohort with varying RNA modification patterns [22, 23]. This was done to highlight significantly enriched biological processes. An enrichment analysis was carried out, with a predefined significance threshold set at a *P* value < 0.05.

GSEA is employed as an analytical technique to determine whether a preselected group of genes exhibits significant differences between two distinct biological conditions. This method is commonly used to identify variations in pathway and biological function involvement within gene expression datasets [29]. To elucidate the differences in biological processes among patients with distinct RNA modification patterns, gene expression profile datasets were obtained. Following this, reference gene collections “c5.go.v7.4.entrez.gmt” and “C2.cp.keg.v7.4.Entrez.gmt” were sourced from the MSigDB database [30]. The GSEA protocol, integrated into the R package cluster Profiler (version 2.1.6), facilitated the enrichment analysis and subsequent data visualization. An adjusted *P* value < 0.05 was considered indicative of statistical significance.

GSVA, a non-parametric unsupervised method, primarily enables the conversion of gene expression matrices from various samples into corresponding matrices for gene sets. This allows for an assessment of transcriptomic enrichment, revealing potential differential engagement of metabolic pathways across samples [31]. To explore the differences in biological functions among patients characterized by distinct RNA modification profiles, the “GSVA” R package (version 1.50.0) was employed [31]. This facilitated a comprehensive analysis of variations based on the gene expression profiles of the specified patient cohorts. The reference compilation “h.all.v7.4.symbols.gmt” was sourced from the MSigDB repository [30], aiding in the determination of enrichment scores for each patient per hallmark within the dataset. Subsequently, an analysis was conducted to identify correlations among dysregulated pathways within the patient population. A predefined criterion of *P* value < 0.05 indicated statistical significance.

### Interplay Within PPI Framework

The gene expression landscape is known for its complexity, often involving the collaboration of specific genes, especially when they regulate similar biological pathways. To decipher these associations among patients characterized by distinct RNA modification patterns, PPI networks were constructed based on their DEGs. The STRING repository was utilized [32], setting a stringent comprehensive score of 700 as the threshold for creating PPI networks around key RNA-modified genes in MDD patients. This constructed network was subsequently imported into Cytoscape for further analysis [33].

Within Cytoscape’s Cytohubba plugin, 12 computational methods (including betweenness, bottleneck, closeness, clustering coefficient, degree, DMN, eccentricity, EPC, MCC, MNC, radiality, and stress [34]) were employed to identify the top 30 hub nodes in each method. Genes identified in at least five of these methods were designated as central hubs. Due to their extensive interconnectedness, these pivotal hubs are believed to exert significant influence over the overall biological processes, warranting in-depth exploration.

MicroRNAs (miRNAs), inherently non-coding and single-stranded RNA molecules encoded within our genome, play crucial roles in various biological processes, including tumorigenesis, biological growth, organogenesis, and epigenetic regulation, as well as defense against viral entities. The regulatory networks involving miRNAs are complex, as a single miRNA can regulate numerous target genes, while a specific gene can be targeted by several miRNAs [35]. To gain deeper insights into the core genes and their interactions with microRNAs, we identified associated miRNAs for these hub genes using the Starbase platform. Starbase utilizes a combination of seven prediction tools (including miRmap, RNA22, microT, PITA, picTar, miRNAda, and TargetScan) to provide insights into potential miRNA-gene connections. By requiring confirmation from at least two of these algorithms, we enhanced our understanding of miRNA-mRNA interactions, resulting in the creation of a complex mRNA-miRNA regulatory network. This network was then visualized using Cytoscape for better accessibility.

Transcription factors (TFs), by nature, regulate gene expression by forming associations with specific target genes. To elucidate the regulatory influence exerted by these core genes, we obtained TF-to-hub gene connections from the miRNet repository. This allowed us to establish a comprehensive interaction framework encompassing both hub genes and TFs. Once again, for improved accessibility and comprehension, this interaction network was visualized using the Cytoscape tool.

### Determination and Associative Analysis of Immune Cells’ Infiltration Based on RNA Modification Diversities

Utilizing the ssGSEA methodology, the prevalence of 28 distinct immune cell types was quantified in subjects with varying RNA modification profiles [30]. Subsequently, within the R environment [29], the representation of 22 specific immune cells was assessed among patients, each characterized by unique RNA modification characteristics in the dataset, using the CIBERSORT algorithm.

### Analytical Methodology

The R software suite (version 4.1.1) was utilized for the computational analysis and subsequent data processing. To assess continuous variables between the paired cohorts, the independent *t*-test was employed to determine the significance level of normally distributed variables. For non-normally distributed variables, the Wilcoxon rank-sum test was employed to compare independent variables between these groups. To measure the degree of association between different genes, the Pearson correlation method was applied. ROC curves were generated using a dedicated R package (Project home page: http://expasy.org/tools/pROC/), with both ROC and AUC measurements serving as metrics for assessing diagnostic accuracy [36]. All calculated *P*-values were two tailed, with a threshold of *P* < 0.05 used as the criterion for statistical significance.

### Analytical Methodology for Hub Gene Validation

Comprehensive information regarding the central genes, including nomenclature, symbolic representations, and biological roles, was obtained from the National Center for Biotechnology Information (NCBI) repository. Subsequently, the differential expression of eight crucial genes was determined by conducting qRT-PCR on blood specimens from a cohort consisting of six individuals diagnosed with MDD and an equal number of matched controls.

The initial step involved the extraction of total RNA from these specimens, following the protocol provided, using the RNeasy Plus Mini Kit (QIAGEN). This was followed by cDNA synthesis, which was facilitated by the TransScript All-in-One First-Strand cDNA Synthesis SuperMix (TRANSGEN). The thermal regimen included an initial phase at 25 °C for 5 min, a secondary phase at 55 °C for 15 min, and a final phase at 85 °C for 5 min. Subsequently, the amplification of this synthesized cDNA was achieved using the PerfectStartTM Green qPCR SuperMix (TRANSGEN) with a thermal cycling protocol consisting of an initial step at 94 °C for 30 s, followed by 40 iterative cycles at 94 °C for 5 s, and a final elongation step at 60 °C for 30 s. The differential expression of these eight genes of interest was quantified using the 2 − △△Ct method, with GAPDH serving as the reference gene (Table 2).

**Table 2:** Primer sequences

Gene	Forward primer (5′–3′)	Reverse primer (5′–3′)
ZC3H13	CGGACACTAACTCCACCTTTAC	TCCCTAGTATCTCTGGCATCTC
YTHDC2	GTGGCAGGCATGTATCCTAAT	TTCTATGGGCTCTGGTCATTTC
TRMT112	CATGAAACTGCTTACCCACAATC	GGTCCTCAGAAACTCCTCATTC
MBD3	CCTGTCTCTATCTCTCCCTCTT	CCTCTAGCAAAGGCCAGTATT
TRMT6	AAGAAGCGGGCACTGATAAT	TCTGGGCTAGTGTATCGTATCT
IGF2BP1	GGGATTAGGGTGTGGTGTTT	CAGTTTGGCAGAGGGTATGT
NUDT21	GTAAGTACGTGAGCCAGTCATC	AGTGCCCTTATACCCTCTTCTA
XRN1	CGAGGCACCATCATAGGAATAA	GCCCAGAGGAAACTGATGAA
GAPDH	GTATCGTGGAAGGACTCATGAC	ACCACCTTCTTGATGTCATCAT

## Results

### Analysis of Gene Transcription Pertaining to RNA Modification in MDD-afflicted Patients

**Fig. 2:**
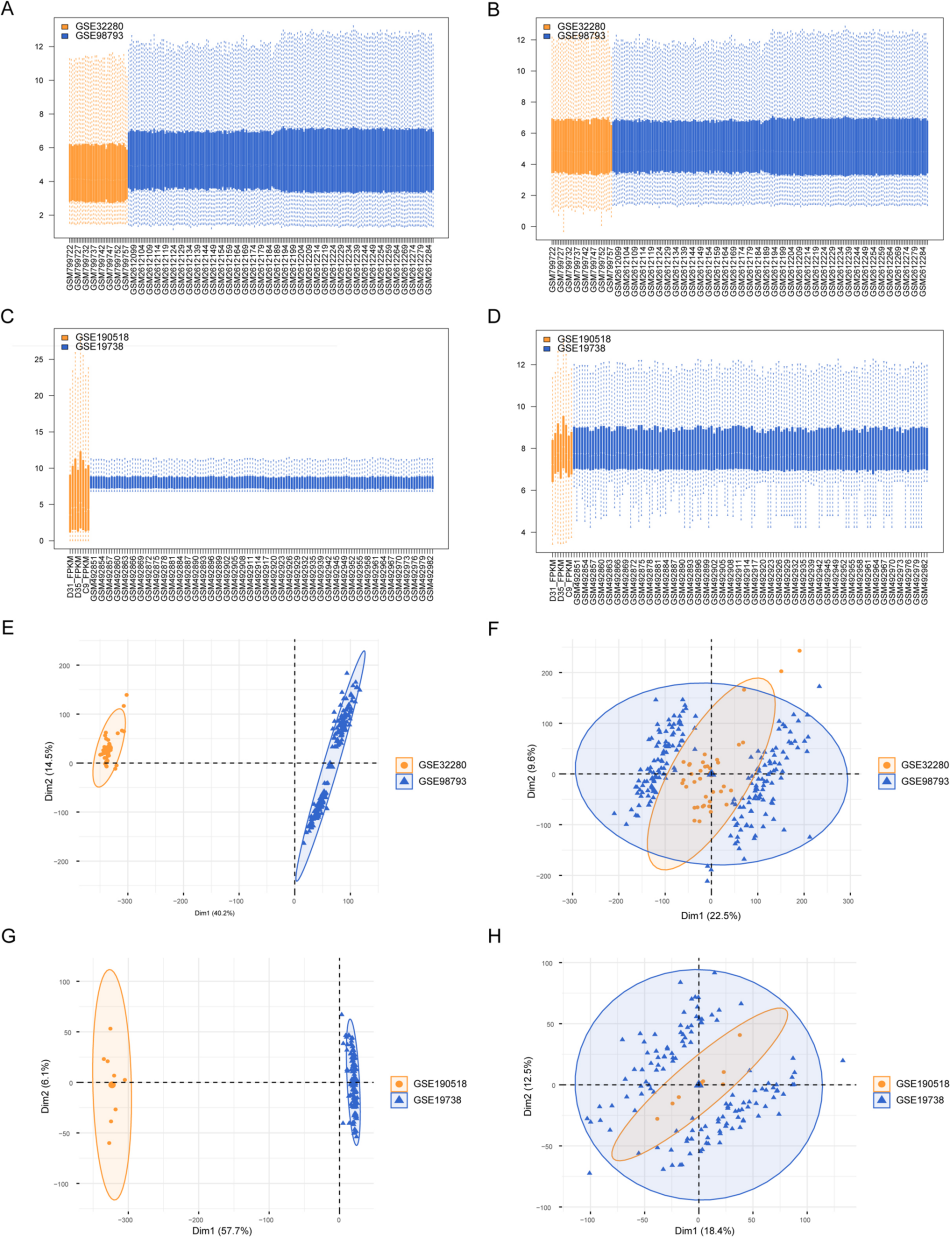
Data set integration. **A**, **C** Integrated sample gene expression level plot; horizontal axis is the sample and vertical axis is the gene expression level. **B**, **D** Gene expression level plot of integrated samples after removing batch effect; horizontal axis is sample and vertical axis is gene expression level. **E**, **G** Cluster plots of samples before removing batch effects. **F**, **H** Sample clustering plots after removing batch effects

The initial phase involved the integration of datasets from GSE32280 and another unspecified dataset, denoted as GSE98793 (Fig. 2A). This amalgamation revealed significant batch discrepancies between the two datasets (Fig. 2E). By addressing and rectifying these batch effects, a consistent gene transcription profile was generated, as elucidated in Fig. 2B. The consolidated dataset comprised transcriptional data from 144 MDD specimens and 72 controls, as represented in Fig. 2F.

Subsequently, an integration was performed with GSE19738 and another unspecified dataset, GSE190518 (Fig. 2B). This combination also exhibited prominent batch inconsistencies between the datasets, as observed in Fig. 2G. Upon addressing these discrepancies, a harmonized gene transcription profile emerged (Fig. 2D). This curated dataset included 41 samples from MDD-afflicted patients and 39 from control cohorts (Fig. 2H).

**Fig. 3:**
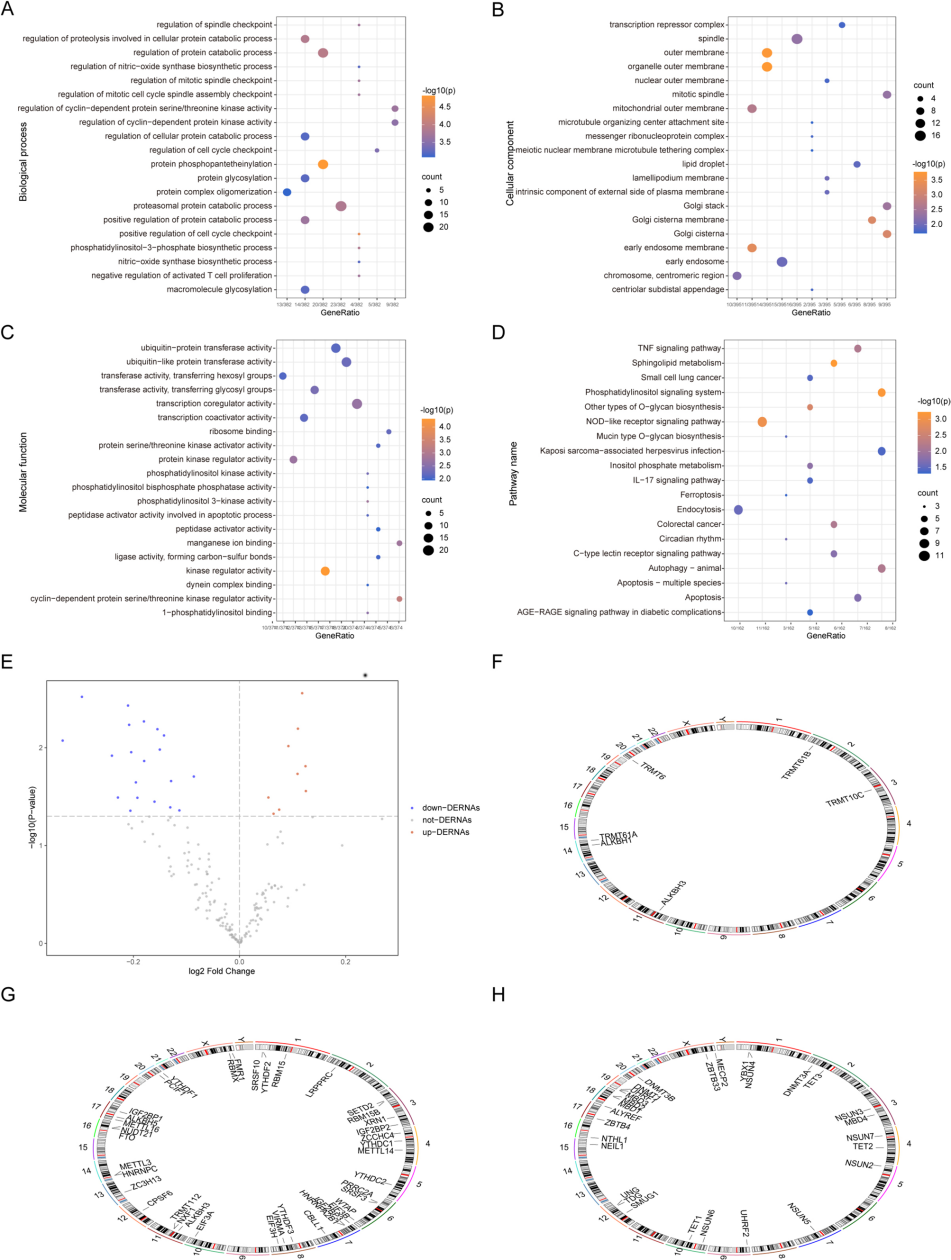
Functional enrichment analysis of differentially expressed genes. **A**–**D** BP, CC, MF analysis and KEGG enrichment analysis in GO terms of differentially expressed genes related to depression; horizontal axis is generation, vertical axis is GO terms, node size indicates the number of genes included in the current GO term, and node color indicates significance level. **E** MDD-related differentially expressed RNA modified genes volcano plot abscise is log2 fold change, ordinate is − log10(*P* value), red nodes represent up-regulated differentially expressed genes, blue nodes represent down-regulated differentially expressed genes, and gray nodes represent genes that are not significantly differentially expressed. **F**, **G** Distribution of m1A-, m5C-, and m6A-related RNA modification-related genes in chromosomes. MDD, major depressive disorder; BP, biological process; CC, cellular component; GO, Gene Ontology; KEGG, Kyoto Encyclopedia of Genes and Genomes; MF, molecular function

In our analysis, 450 DEGs were identified when comparing MDD samples with controls. Among these, 23 DEGs exhibited increased expression, while 427 showed diminished expression. Regarding GO classifications, these DEGs were predominantly associated with processes such as protein phosphopantetheinylation, cell cycle checkpoint regulation, and modulation of protein degradation pathways (Fig. 3A). At the cellular level, these genes were localized to compartments like the outer organelle membrane, primary outer membrane, and early endosome membranes (Fig. 3B). Functionally, these genes played key roles in kinase regulatory mechanisms, cyclin-driven protein serine/threonine kinase modulation, and phosphatidylinositol 3-kinase functions (PI3KAKT), among others (Fig. 3C). Additionally, KEGG pathway analysis indicated significant enrichment in areas such as sphingolipid metabolism, phosphatidylinositol signal transduction, and pathways associated with NOD-like receptor signaling. The analysis of RNA-modification-related DEGs resulted in the identification of 29 uniquely expressed genes (Fig. 3E), with 9 exhibiting increased expression and 20 demonstrating reduced expression (Fig. 3D). The chromosomal localization of these RNA modification-linked genes was annotated using the RCircos package in R [37], revealing a notable clustering of these genes in analogous chromosomal regions (Fig. 3F–H).

### Risk Model Construction

**Fig. 4:**
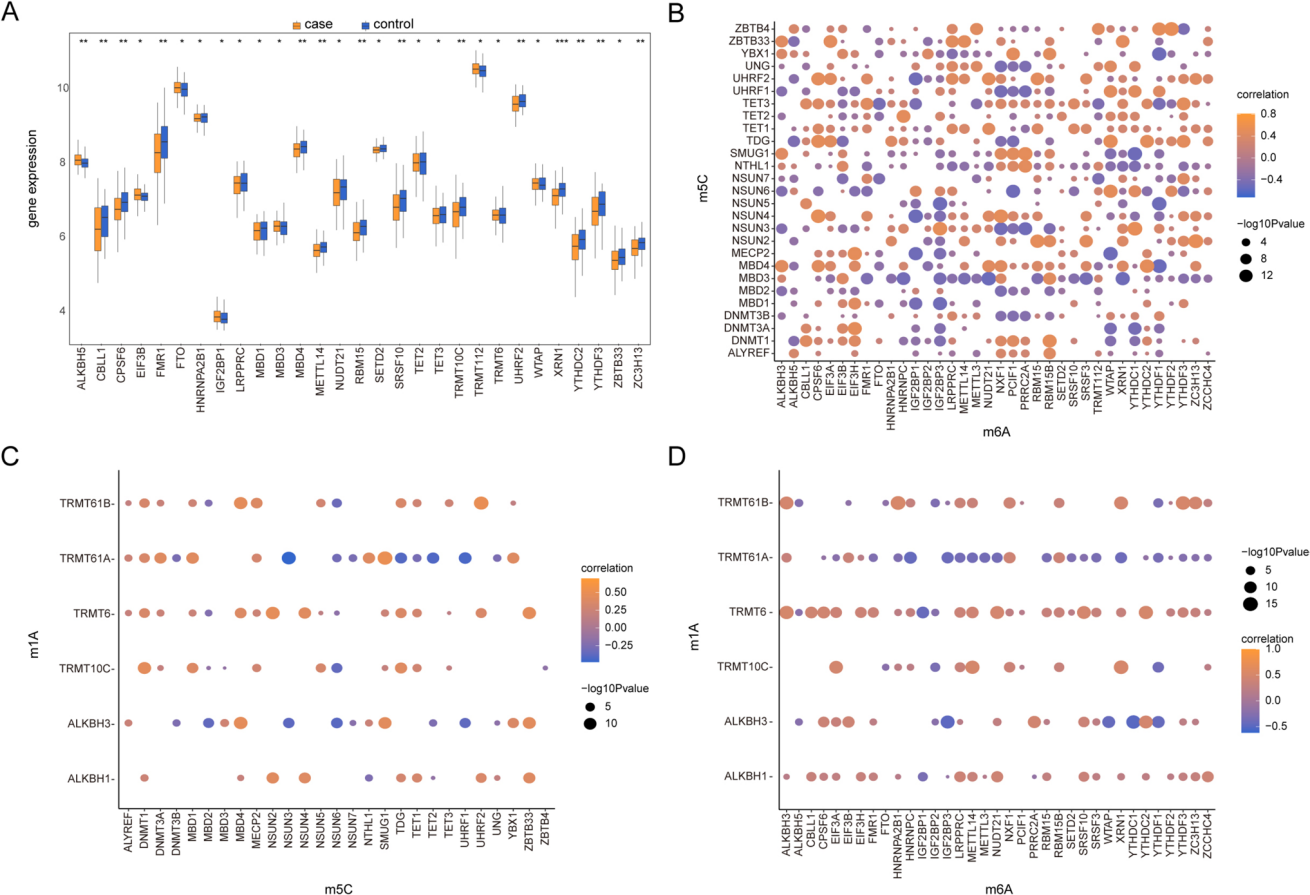
Correlation analysis. **A** The difference in expression levels of depression-related differentially expressed RNA modifier genes between MDD samples and control samples; the horizontal axis is the depression-related differentially expressed RNA modifier genes and the vertical axis is the gene expression levels. **B** Correlation analysis of gene expression levels of RNA modification-related genes related to m5C and m6A in all samples; node size indicates significance, and line node color indicates correlation. **C** Correlation analysis of gene expression levels of RNA modification-related genes related to m5C and m1A in all samples; node size indicates significance and node color indicates correlation. **D** Correlation analysis of gene expression levels of RNA modification-related genes related to m1A and m6A in all samples; node size indicates significance and line node color indicates correlation. MDD, major depressive disorder; * denotes significance less than 0.05; ** denotes significance less than 0.01; and **** denotes significance less than 0.001

Epigenetic modifications of RNAs are increasingly recognized for their roles in various biological functions. Differential gene expression analysis was conducted between MDD and normal samples (Fig. 4A), encompassing RNA modifier genes related to m1A, m6A, and m5C. Subsequently, the expression level correlations among RNA epigenetically modified genes, as well as among the m1A, m6A, and m5C gene sets in all samples, were analyzed separately. The results revealed significant negative correlations between YTHDC1 and NSUN5, PRRC2A, and TDG (*P* < 0.05, Fig. 4B), while NXF1 and MECP2, NXF1, and NSUN5 exhibited strong positive correlations (*P* < 0.05, Fig. 4B). Additionally, a robust positive association was identified between ZBTB33 and TRMT10C, as well as between NSUN5 and ALKBH3 (*P* < 0.05, Fig. 4C). Conversely, pronounced negative relationships were observed between X3 and XA, and between UHRF1 and TRMT61A (*P* < 0.05, Fig. 4C). Moreover, a significant positive linkage was detected between NSUN3 and TRMT61A, and between UHRF1 and TRMT61A (*P* < 0.05, Fig. 4D). In contrast, marked negative interconnections were observed between SRSF10 and TRMT61B, and between NXF1 and TRMT61A (*P* < 0.05, Fig. 4D).

**Fig. 5:**
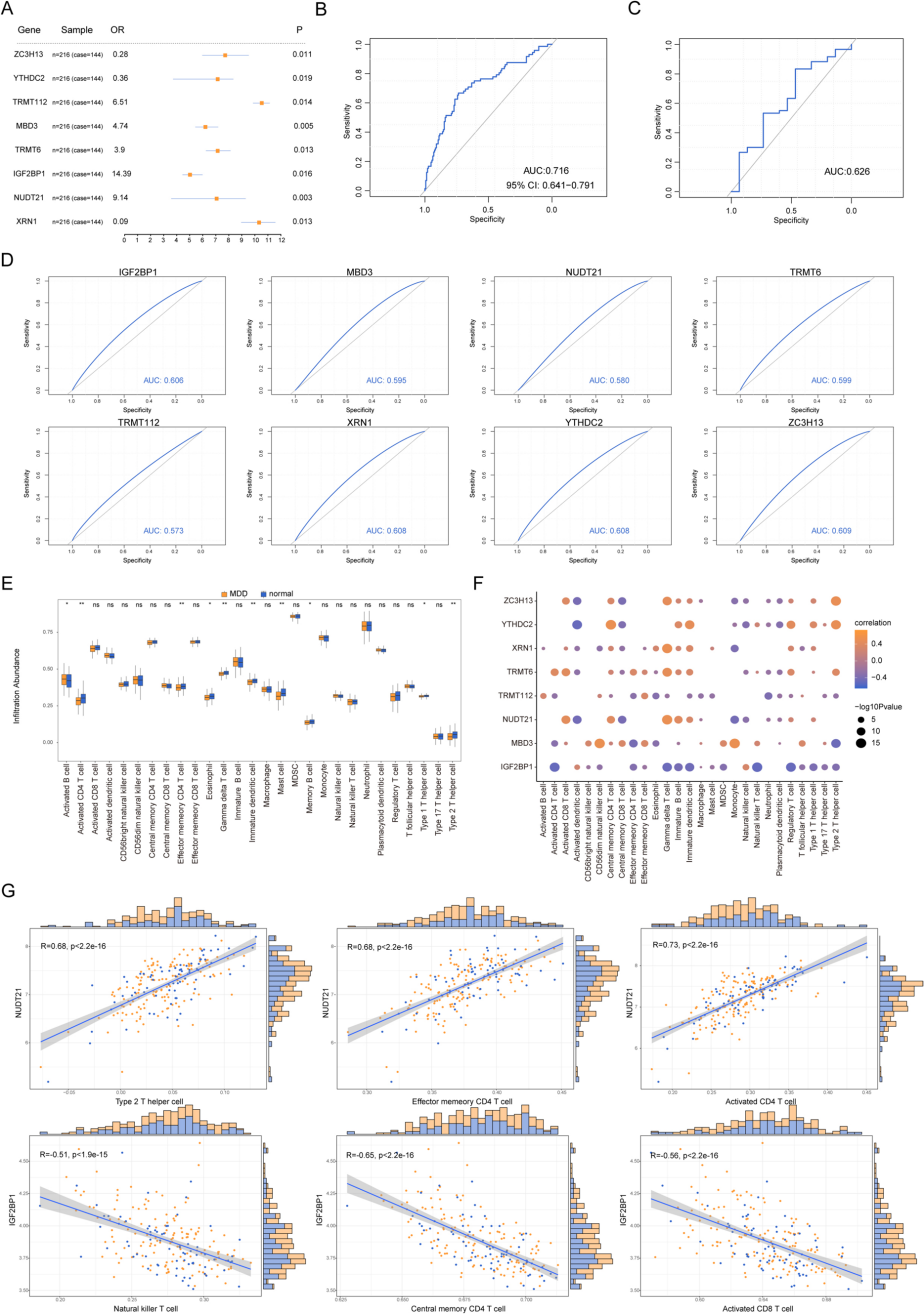
Construction of the depression model. **A** Forest plot of signature genes in patients with depression. **B** ROC 10.1007/s12035-024-04042-6 curve for predicting risk scores in depression training set diagnosis. **C** ROC curve for predicting risk score in depression test set diagnosis. **D** ROC curves for the eight signature genes in the diagnosis of depression. **E** The enrichment box plot of immune cells in control samples and MDD samples; the horizontal axis is immune cells, the vertical axis is immune score, orange represents MDD, and blue represents control group. **F** Correlation between characteristic gene expression level and immune cell content; node size indicates significance and node color indicates correlation. The horizontal axis is the immune cell, and the vertical axis is the characteristic gene. **G** The first three relationship pairs of positive and negative correlations, with immune cells on the horizontal axis and characteristic genes on the vertical axis. ROC, receiver operating characteristic curve; AUC, represents the area under the curve; MDD, major depressive disorder. * represents significance less than 0.05, ** represents significance less than 0.01, and **** represents significance less than 0.001

To explore the impact of genes associated with RNA modifications on MDD, a generalized linear model function in R was employed to identify 8 hallmark genes out of the 29 differentially expressed RNA-modifying genes. These identified genes were labeled as ZC3H13, YTHDC2, TRMT112, MBD3, TRMT6, IGF2BP1, NUDT21, and XRN. Utilizing the coefficients derived from the glm function for these eight pivotal genes (Fig. 5A), gene expression was multiplied by the corresponding coefficients, establishing a prognostic risk metric for MDD. Subsequently, the ultimate prognostic risk index for each sample was computed. Using this risk metric, an ROC analysis was conducted, revealing an AUC of 0.716 for the training dataset (Fig. 5B) and an AUC of 0.626 for the validation set (Fig. 5C). These results indicate the model’s commendable capacity to distinguish MDD cases. Furthermore, when analyzing the ROC curves of these 8 hallmark genes for MDD prediction individually, the data suggested that each of these genes exhibited robust prognostic potential (Fig. 5D).

To assess variations in immune cell infiltration levels between the control and MDD specimens, comprehensive ssGSEA was performed on both sets. Notably, compared to controls, MDD specimens displayed significantly reduced infiltration of immune cells such as eosinophils and gamma delta T lymphocytes (Fig. 5E). Subsequently, correlations between the expression levels of these immune cells and the hallmark genes were computed. Intriguingly, IGF2BP1 gene expression inversely correlated with numerous immune cell types (*r* < 0, *P* < 0.05, Fig. 5F). Most prominently, a direct association was discerned between NUDT21 and immune cells like type 2 T helper cells, effector memory CD4 T cells, and activated CD4 T cells (*P* < 0.05, Fig. 5G). Conversely, IGF2BP1 exhibited the most pronounced inverse relationship with natural killer T cells, central memory CD4 T cells, and activated CD8 T cells (*P* < 0.05, Fig. 5G).

**Fig. 6:**
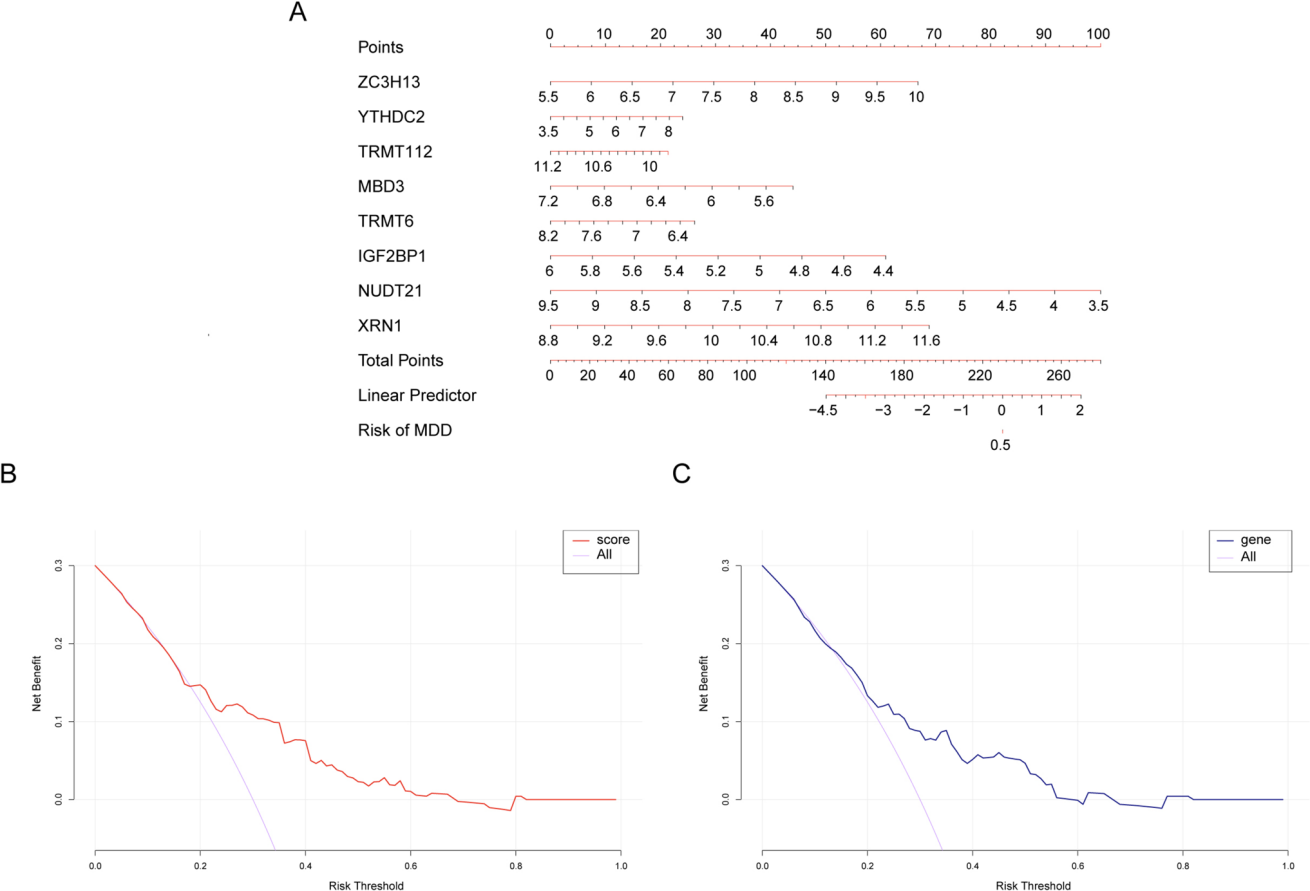
Line and column diagram (nomogram). **A** Nomogram of the eight signature genes for the diagnosis of patients with depression. **B** Model evaluation curve; gray for immediate diagnosis and orange for risk score model. **C** Model evaluation curve; gray indicates immediate diagnosis and blue indicates signature gene combination

A nomogram, incorporating both the predicted risk score and the eight significant genes, was developed to predict the incidence of depression (Fig. 6A). Notably, the anticipated risk score demonstrated substantial predictive capability. Within the decision curve analysis (DCA), the prognostic trajectory consistently outperformed the reference (purple line), suggesting that clinical decisions guided by this nomogram may offer therapeutic advantages for individuals dealing with depression (Fig. 6B, C).

### Identification of Two Unique RNA Modification Profiles

**Fig. 7:**
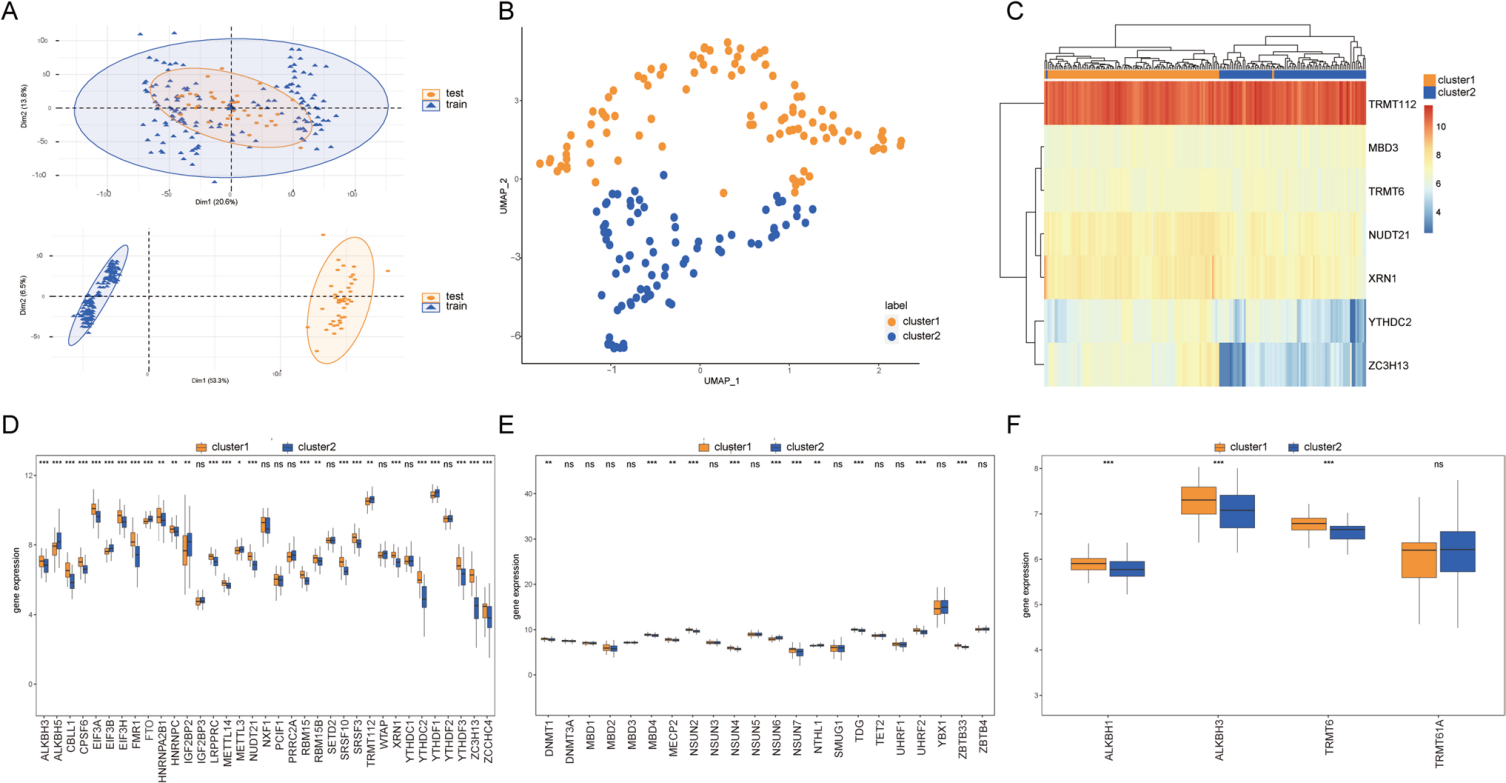
Consistent clustering of feature genes for patients with depression. **A** PCA plot before and after debatching; the horizontal axis and the vertical axis are the two principal components, respectively, orange represents the training set, blue represents the test set, the top panel is PCA after debatching, and the bottom panel is PCA before debatching. **B** UMAP clustering result plot; orange denotes cluster1 and blue denotes cluster 2. **C** Heat map of the expression levels of the feature genes in the two clusters; orange for cluster1 and blue for cluster 2. **D** Difference in m6A expression levels between cluster 1 and cluster 2 samples; orange denotes cluster 1 and blue denotes cluster 2, horizontal axis is the characteristic gene, and vertical axis is the gene expression level. **E** Difference in m5C expression levels between cluster1 and cluster2 samples; orange indicates cluster 1, blue indicates cluster 2, horizontal axis is the characteristic gene, and vertical axis is the gene expression level. **F** Difference in m1A expression levels between cluster1 and cluster 2 samples; orange indicates cluster 1, blue indicates cluster 2, horizontal axis is the characteristic gene, and vertical axis is the gene expression level. PCA, principal component analysis; UMAP, uniform manifold approximation and projection

To increase the sample size of MDD, the test set and training set data were combined using the R package sva (Fig. 7A), resulting in a total of 182 MDD samples. Two distinct RNA modification archetypes, labeled as cluster 1 and cluster 2, were identified using the octet of RNA-modification-associated genes through the UMAP analytical technique (Fig. 7B). Cluster 1 encompassed 101 specimens, while cluster 2 included 81 specimens. An examination of these clusters revealed significant differences in the signature genes between the two clusters (Fig. 7C). The expression patterns of m1A, m5C, and m6A affiliated genes were cataloged across these RNA modulation archetypes. The evaluation revealed marked differential expression of the majority of signature genes associated with RNA modification in both clusters (Fig. 7D–F).

### Functional and Network Analysis of RNA Modification Profiles

**Fig. 8:**
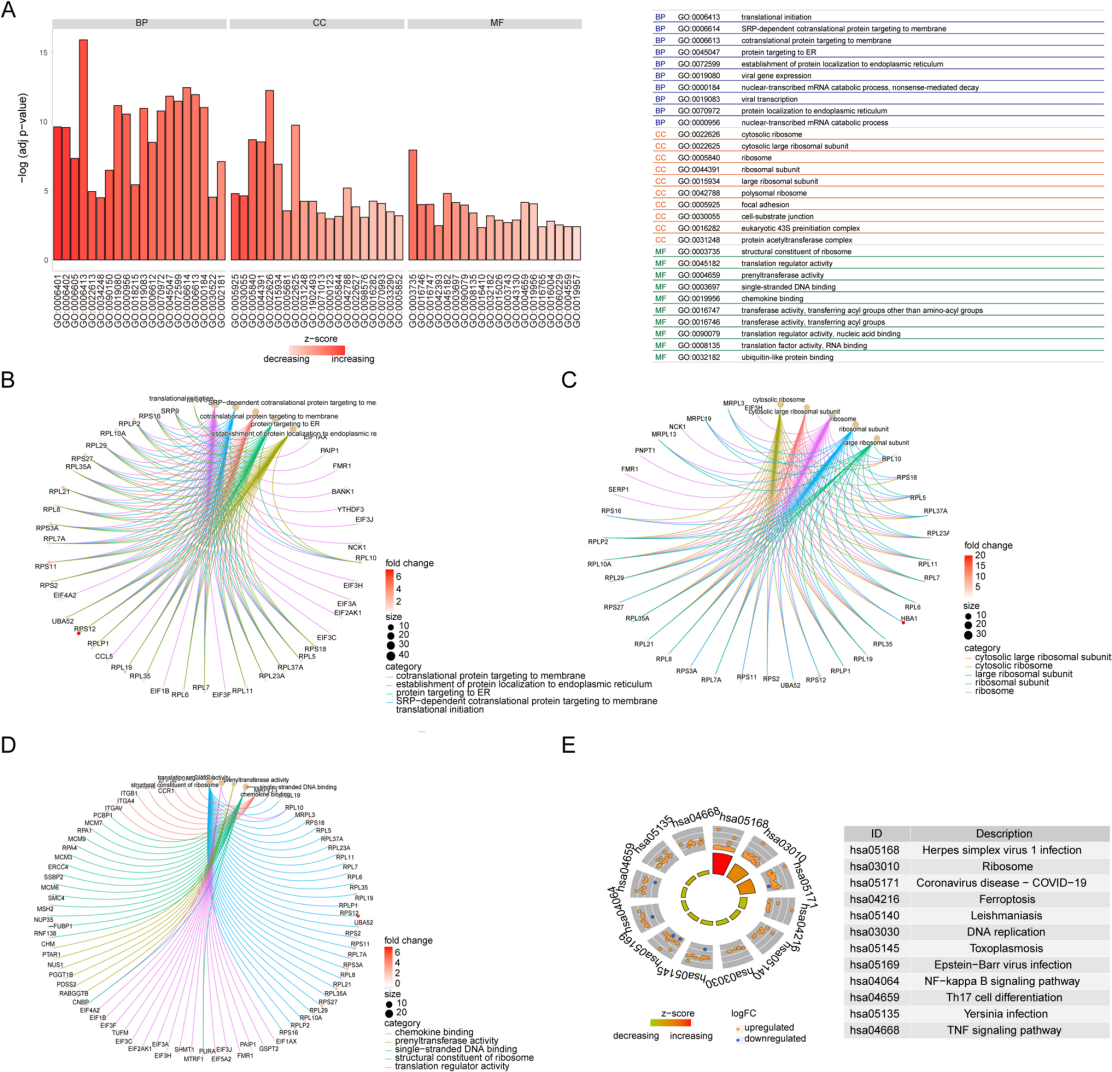
Functional analysis of differentially expressed genes. **A** GO functional enrichment analysis of differentially expressed genes, the ordinate is the significance of enrichment results, and the abscissa is the *Z*-score. **B**–**D** The first five items of BP, CC, and MF are displayed, node size indicates the number of currently functionally enriched genes, and the color of the line indicates different biological functions. **E** KEGG pathway enrichment analysis results; node color indicates the expression level of the gene and quadrangle color indicates the KEGG pathway *Z*-score. BP, biological process; CC, cellular component; GO, Gene Ontology; KEGG, Kyoto Encyclopedia of Genes and Genomes; MF, molecular function

To identify variances in biological processes among individuals with two distinct RNA modification profiles, an initial comparative evaluation of gene expression dynamics was conducted, resulting in the identification of 898 DEGs. A comprehensive GO categorization of these DEGs highlighted their roles in various cellular operations (Fig. 8A, Table 3). These genes were notably enriched in processes such as protein synthesis initiation, membrane-associated SRP-mediated cotranslational protein localization, and protein transport to the endoplasmic reticulum (Fig. 8B). At the cellular level, they were associated with cytosolic ribosomal assemblies and larger ribosomal fractions (Fig. 8C). Additionally, these genes were significant in modulating ribosomal structure, regulating translation, and facilitating prenyltransferase functionalities (Fig. 8D). Pathway enrichment analysis revealed their involvement in key biological pathways, including responses to Herpes simplex virus 1, ribosomal architecture, and pathways related to Coronavirus disease (Fig. 8E, *P* < 0.05, Table 4).

**Table 3:** GO enrichment analysis

Category	ID	Description	*P* value
BP	GO:0006413	Translational initiation	1.24E − 16
BP	GO:0006614	SRP-dependent cotranslational protein targeting to membrane	3.50E − 13
BP	GO:0006613	Cotranslational protein targeting to membrane	1.13E − 12
BP	GO:0045047	protein targeting to ER	1.46E − 12
BP	GO:0072599	Establishment of protein localization to endoplasmic reticulum	3.36E − 12
BP	GO:0019080	Viral gene expression	6.96E − 12
BP	GO:0000184	Nuclear-transcribed mRNA catabolic process, nonsense-mediated decay	9.53E − 12
BP	GO:0019083	Viral transcription	1.12E − 11
BP	GO:0070972	Protein localization to endoplasmic reticulum	1.73E − 11
BP	GO:0000956	Nuclear-transcribed mRNA catabolic process	2.85E − 11
MF	GO:0140297	DNA-binding transcription factor binding	1.27E − 10
MF	GO:0003735	Structural constituent of ribosome	1.13E − 08
MF	GO:0045182	Translation regulator activity	1.56E − 05
MF	GO:0004659	Prenyltransferase activity	6.94E − 05
MF	GO:0003697	Single-stranded DNA binding	7.03E − 05
MF	GO:0019956	Chemokine binding	8.84E − 05
MF	GO:0016747	Transferase activity, transferring acyl groups other than amino-acyl groups	9.47E − 05
MF	GO:0016746	Transferase activity, transferring acyl groups	9.80E − 05
MF	GO:0090079	Translation regulator activity, nucleic acid binding	0.000108315
CC	GO:0022626	Cytosolic ribosome	5.72E − 13
CC	GO:0022625	Cytosolic large ribosomal subunit	1.81E − 10
CC	GO:0005840	Ribosome	2.06E − 09
CC	GO:0044391	Ribosomal subunit	2.92E − 09
CC	GO:0015934	Large ribosomal subunit	1.21E − 07
CC	GO:0042788	Polysomal ribosome	6.36E − 06
CC	GO:0005925	Focal adhesion	1.60E − 05
CC	GO:0030055	Cell-substrate junction	2.33E − 05
CC	GO:0016282	Eukaryotic 43S preinitiation complex	5.63E − 05

**Table 4:** KEGG enrichment analysis

Category	ID	Description	*P* value
KEGG_PATHWAY	hsa05168	Herpes simplex virus 1 infection	1.40E − 12
KEGG_PATHWAY	hsa03010	Ribosome	1.48E − 09
KEGG_PATHWAY	hsa05171	Coronavirus disease—COVID-19	1.65E − 08
KEGG_PATHWAY	hsa04216	Ferroptosis	0.000112847
KEGG_PATHWAY	hsa05140	Leishmaniasis	0.001066957
KEGG_PATHWAY	hsa03030	DNA replication	0.001414174
KEGG_PATHWAY	hsa05145	Toxoplasmosis	0.002730708
KEGG_PATHWAY	hsa05169	Epstein-Barr virus infection	0.003911116
KEGG_PATHWAY	hsa04064	NF-kappa B signaling pathway	0.004120469
KEGG_PATHWAY	hsa04659	Th17 cell differentiation	0.005595246

**Fig. 9:**
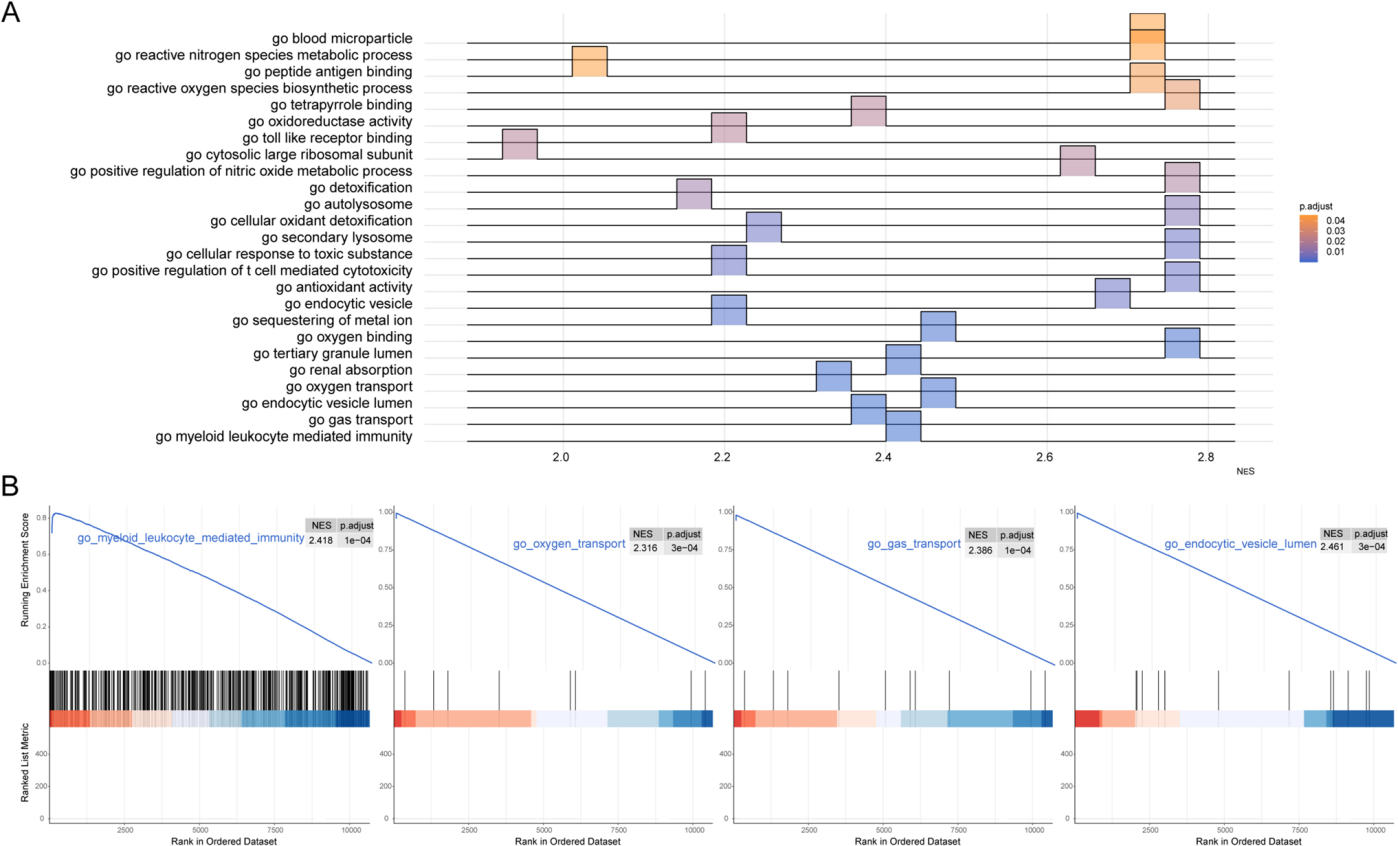
GSEA analysis. **A** GSEA-GO analysis; the abscissa is the enrichment score, a score greater than 0 indicates activation, the ordinate is the GO terms, and the color table *P* value. **B** Top 4 GO terms presented. GSEA, gene set enrichment analysis; GO, Gene Ontology

Subsequently, GSEA was performed on individuals representing the two distinct RNA modification patterns, revealing that most of these genes exhibited elevated expression levels primarily in the cluster1 cohort, thereby enhancing numerous cellular and metabolic activities. Specifically, the analysis highlighted their pivotal roles in processes such as the biosynthesis of reactive oxygen species, peptide antigen affinities, metabolism associated with reactive nitrogen species, and the dynamics of microparticles within blood plasma (Fig. 9, Table 5).

**Table 5:** GSEA enrichment analysis

ID	ES	NES	*P* value
go_myeloid_leukocyte_mediated_immunity	0.828058	2.418731	1.41E − 08
go_gas_transport	0.993889	2.386485	3.60E − 08
go_endocytic_vesicle_lumen	0.994225	2.461468	1.57E − 07
go_oxygen_transport	0.994563	2.316307	2.05E − 07
go_renal_absorption	0.993434	2.412077	3.22E − 07
go_tertiary_granule_lumen	0.981281	2.755318	6.11E − 07
go_oxygen_binding	0.992154	2.456341	7.33E − 07
go_sequestering_of_metal_ion	0.950524	2.21374	2.55E − 06
go_endocytic_vesicle	0.90377	2.676805	7.99E − 06
go_antioxidant_activity	0.977022	2.753334	8.14E − 06
go_positive_regulation_of_t_cell_mediated_cytotoxicity	0.86509	2.211771	8.80E − 06
go_cellular_response_to_toxic_substance	0.964193	2.77193	9.11E − 06
go_secondary_lysosome	0.902767	2.235041	1.06E − 05
go_cellular_oxidant_detoxification	0.969475	2.763461	2.22E − 05
go_autolysosome	0.928951	2.163498	3.91E − 05
go_detoxification	0.961006	2.77699	5.08E − 05
go_positive_regulation_of_nitric_oxide_metabolic_process	0.980921	2.627245	5.89E − 05
go_cytosolic_large_ribosomal_subunit	0.696208	1.95529	6.96E − 05
go_toll_like_receptor_binding	0.913944	2.194524	7.17E − 05
go_oxidoreductase_activity	0.810987	2.370706	7.28E − 05

**Fig. 10:**
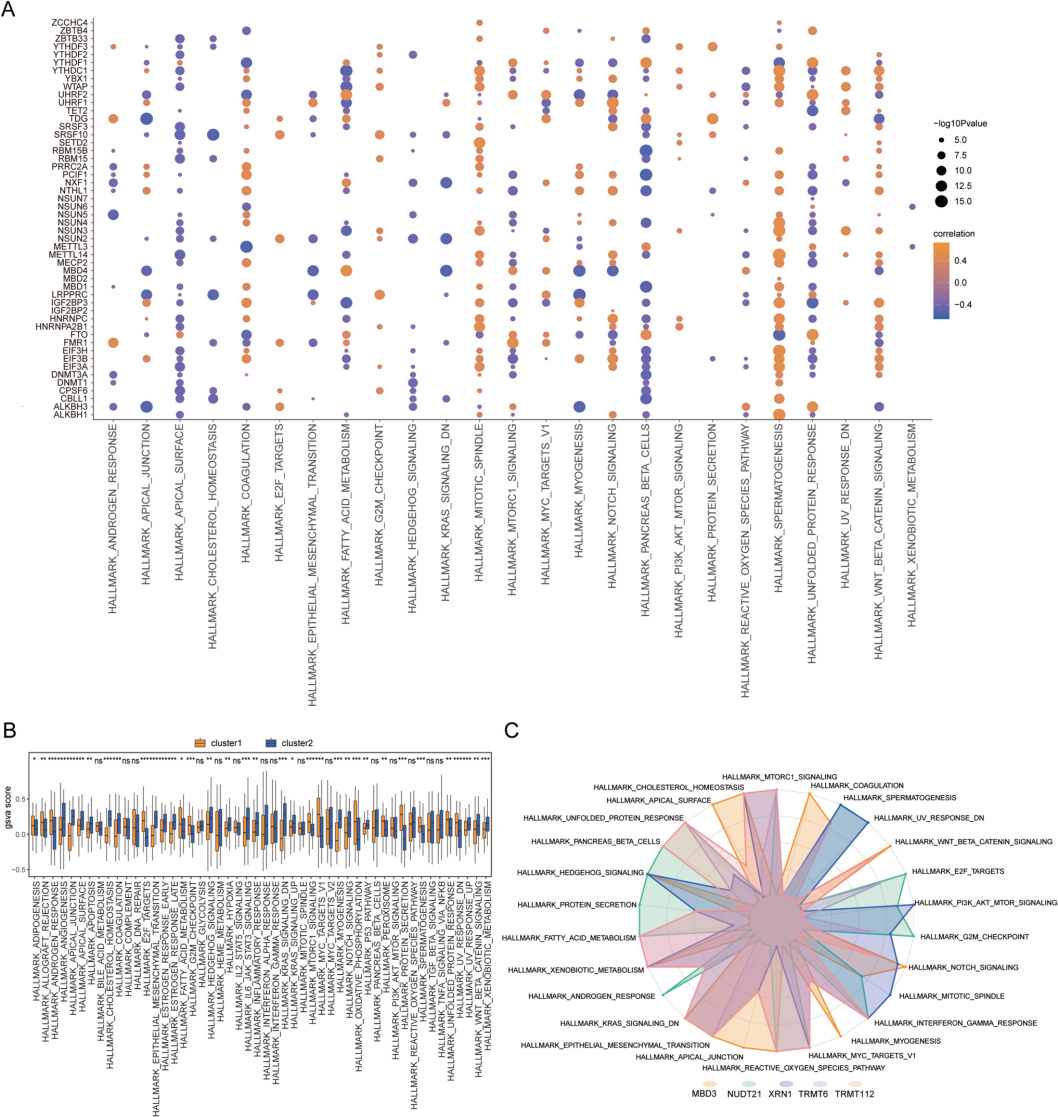
GSVA analysis. **A** Correlation between hallmark and genes related to RNA modification in GSVA analysis; the abscissa is hallmark, the vertical axis is RNA modification related genes, node size indicates significance, and node color indicates correlation. **B** Hallmark difference between patients with two RNA modification patterns, coordinate is hallmark, vertical axis is GSVA score, orange denotes cluster 1, blue denotes cluster 2. **C** Correlation between signature genes and hallmark; the color of the line indicates different signature genes, the node indicates hallmark, and the contour line indicates the level of correlation. GSVA, gene set variation analysis; *, significance less than 0.05; **, significance less than 0.01; ****, significance less than 0.001

GSVA was then conducted to further investigate functional differences in patients with the two RNA modification patterns. The results revealed associations between the expression levels of numerous RNA-modifying genes and biological processes such as apical junction, apical surface, mitotic spindle, and mammalian target of rapamycin complex 1 (mTORC1) signaling (Fig. 10A). In patients exhibiting both RNA modification patterns, notable distinctions were observed in various biological processes, including androgen responsiveness, allograft rejection, adipogenesis, and apical junction dynamics (Fig. 10B). Additionally, we examined the correlation between patient signature genes of both RNA modification patterns and hallmark biological processes. TRMT112 exhibited a notable association with mTORC1 signaling and showed a significant positive correlation with cholesterol homeostasis (*P* < 0.05). Conversely, the unfolded protein response displayed a significant inverse relationship with XRN1 but manifested a pronounced positive affiliation with PI3K AKT mTOR signaling (*P* < 0.05, Fig. 10C).

### Network Analysis of RNA Modification Profiles

**Fig. 11:**
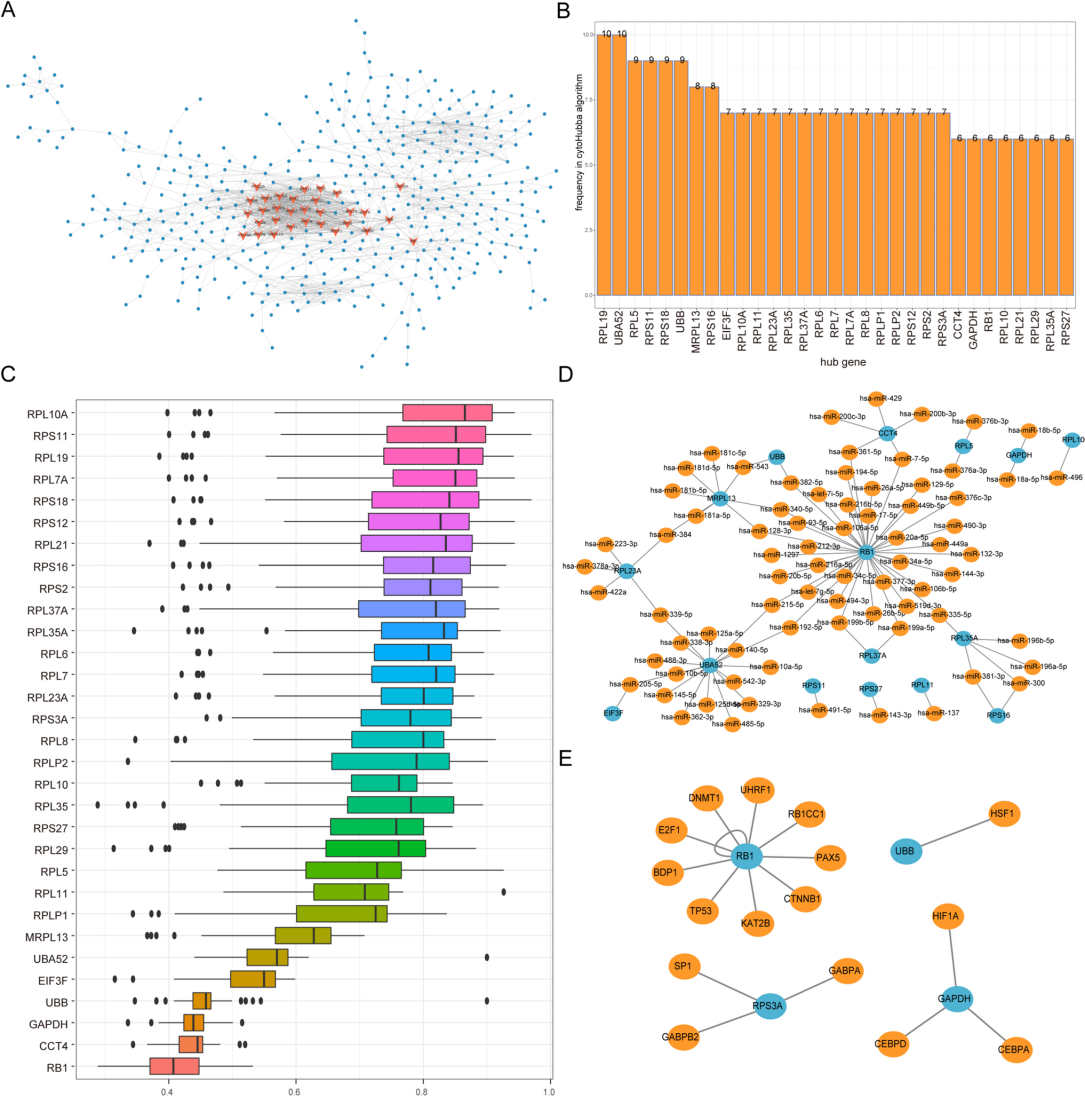
Correlation network of differentially expressed genes. **A** PPI network of differentially expressed genes; blue nodes are differentially expressed genes and orange nodes are hub genes. **B** Gene frequency table in the algorithm in 12; the horizontal axis is the gene and the vertical axis is the frequency. **C** GO semantic similarity score of hub genes in the protein-protein interaction network of differentially expressed genes; horizontal axis is the similarity level and vertical axis is the gene. **D** mRNA-miRNA network of hub genes; blue nodes are hub genes and orange nodes are miRNAs. **E** mRNA-TF network of hub genes; blue nodes are hub genes and orange nodes are TFS. GO, Gene Ontology; PPI, protein-protein interaction; TFS, transcription factors

To evaluate the impact of DEGs in patients with two distinct RNA modification profiles on biologically relevant functions associated with MDD, a PPI network involving these differentially expressed genes was initially established and visualized using Cytoscape. The PPI network comprised 1597 interaction pairs and involved 507 differentially expressed genes. Notably, high node genes included UBA52, RPS2, and RPS11, which interacted with 58, 51, and 50 genes, respectively (Fig. 11A). Using 12 analytical methodologies available in Cytohubba, we systematically assessed and identified the top 30 nodes for each method. Subsequently, 31 genes that appeared in at least five methodologies were identified as central nodes (Fig. 11B).

To explore the functional relevance of these central genes, we utilized the “GOSemSim” package in R to determine their GO semantic similarities [38]. It became evident that genes like RPL19, RPS11, and RPL10A exhibited significant functional associations with numerous other genes (Fig. 11C).

A comprehensive mRNA-miRNA interaction network involving these central genes was constructed, encompassing 91 relational interactions, which involved 16 mRNA sequences and 75 miRNA sequences. Notably, a principal RNA modifying gene, referred to as UBA52, established interactions with 37 miRNAs, while UBA52 formed interactions with 16 miRNAs (Fig. 11D).

Furthermore, an mRNA-TF interaction framework involving the central genes was delineated, comprising 17 interactions that involved 4 mRNAs and 17 TFs. The pivotal transcription factor gene RB1 was identified as it forged interactions with 10 TFs (Fig. 11E).

### Comparative Immune Profiling Across RNA Modification Patterns

**Fig. 12:**
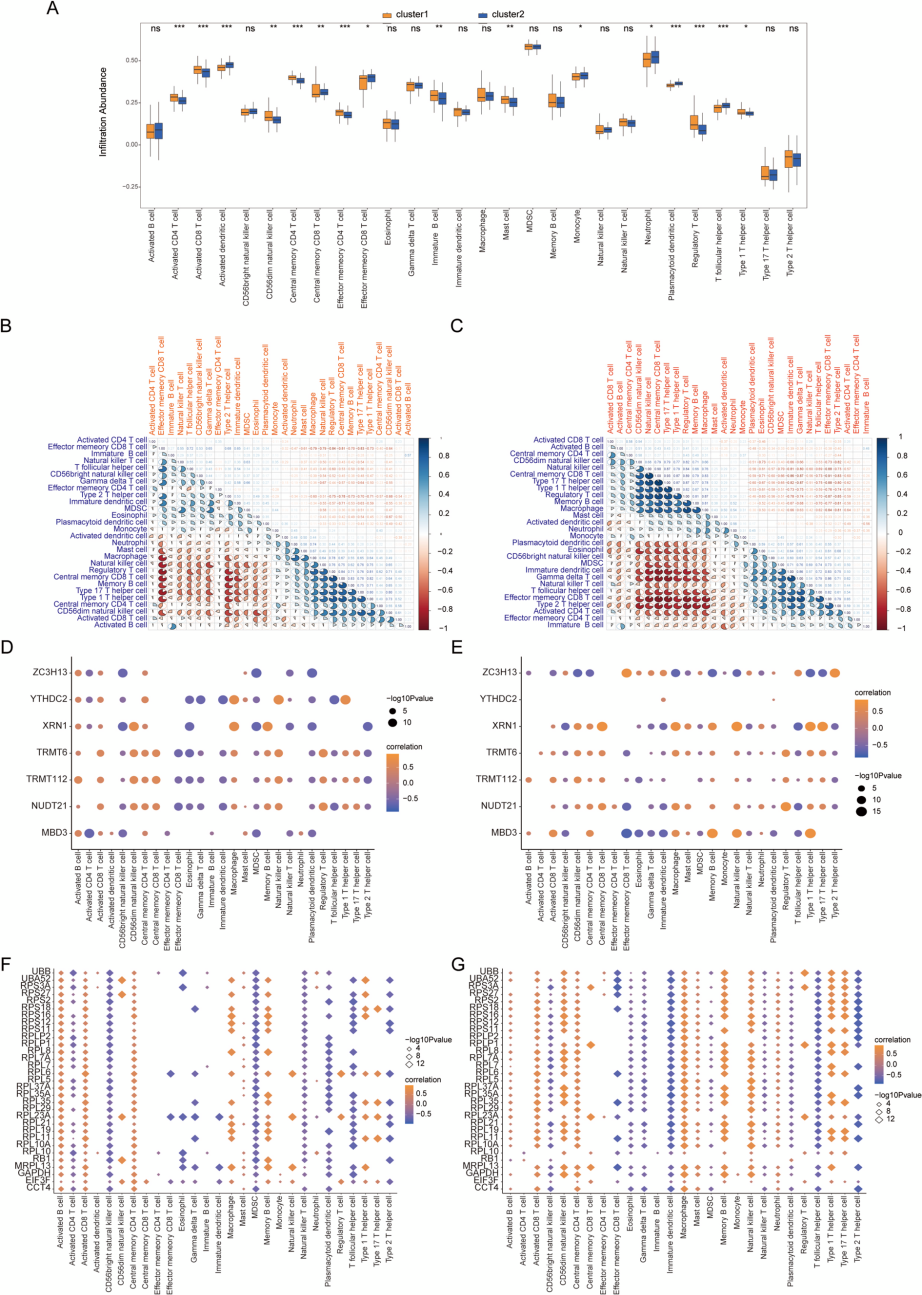
Immune signatures between the two RNA modification patterns ssGSEA. **A** Bar graph of immune cell content in cluster 1 and cluster 2 group patients; blue denotes cluster 2 samples, orange denotes cluster 1 samples, horizontal axis denotes immune cells, and vertical axis denotes cellular content. **B**, **C** Correlation analysis between immune cell content in cluster 1 and cluster 2; red denotes negative correlation and blue denotes positive correlation. **D**–**F** Correlation between feature genes and immune cells in cluster 1 and cluster 2; the horizontal axis 10.1007/s12035-024-04042-6 represents immune cells, the vertical axis represents feature genes, the node color represents the correlation size, and the node size represents the significance level. **G**, **H** Correlation between hub genes and immune cells in cluster 1 and cluster 2; node size indicates significance and node color indicates correlation level. Immune cells are on the horizontal axis and hub genes are on the vertical axis. ssGSEA, single-sample gene set enrichment analysis

The ssGSEA and CIBERSORT computational methods were employed to meticulously assess disparities in immune cell infiltration between the divergent RNA modification frameworks. The insights garnered from ssGSEA indicated that the cluster2 ensemble exhibited a significantly heightened presence of immune constituents, such as activated CD4 T cells and activated CD8 T cells, when compared to the cluster1 cohort (Fig. 12A).

Subsequently, a correlative evaluation of immune cell densities across both groupings was undertaken. Within the cluster1 landscape, a prominent inverse relationship was observed between the presence of activated B cells and the predominance of other cellular entities (Fig. 12B). In contrast, within the cluster2 domain, a multitude of cells, including eosinophils, CD56 luminescent natural killer entities, myeloid-derived suppressor cells (MDSCs), nascent dendritic cells, gamma delta T cells, natural killer T cells, T follicular auxiliary cells, effector memory CD8 T cells, type 2 T helper cells, and activated CD4 T cells, demonstrated discernible negative correlations among themselves (Fig. 12C).

In an effort to elucidate the potential interplay between salient genes and immune cellular entities, an exhaustive correlation assessment was conducted within both the cluster 1 and cluster 2 patient subsets. Surprisingly, within the cluster 1 milieu, gene YTHDC2 exhibited a pronounced association with a vast majority of the immune cells, in stark contrast to its inconspicuous linkage within the cluster 2 demographic (*P* < 0.05, Fig. 12D, E). Furthermore, an intricate analysis was executed to understand the relationship between pivotal genes and immune cells across the two defined clusters. Within the realm of cluster 1, the predominance of activated CD4 T cells demonstrated a substantial negative association with several core genes. Conversely, within the cluster 2 framework, this particular correlation appeared to be marginal (*P* < 0.05, Fig. 12F, G).

**Fig. 13:**
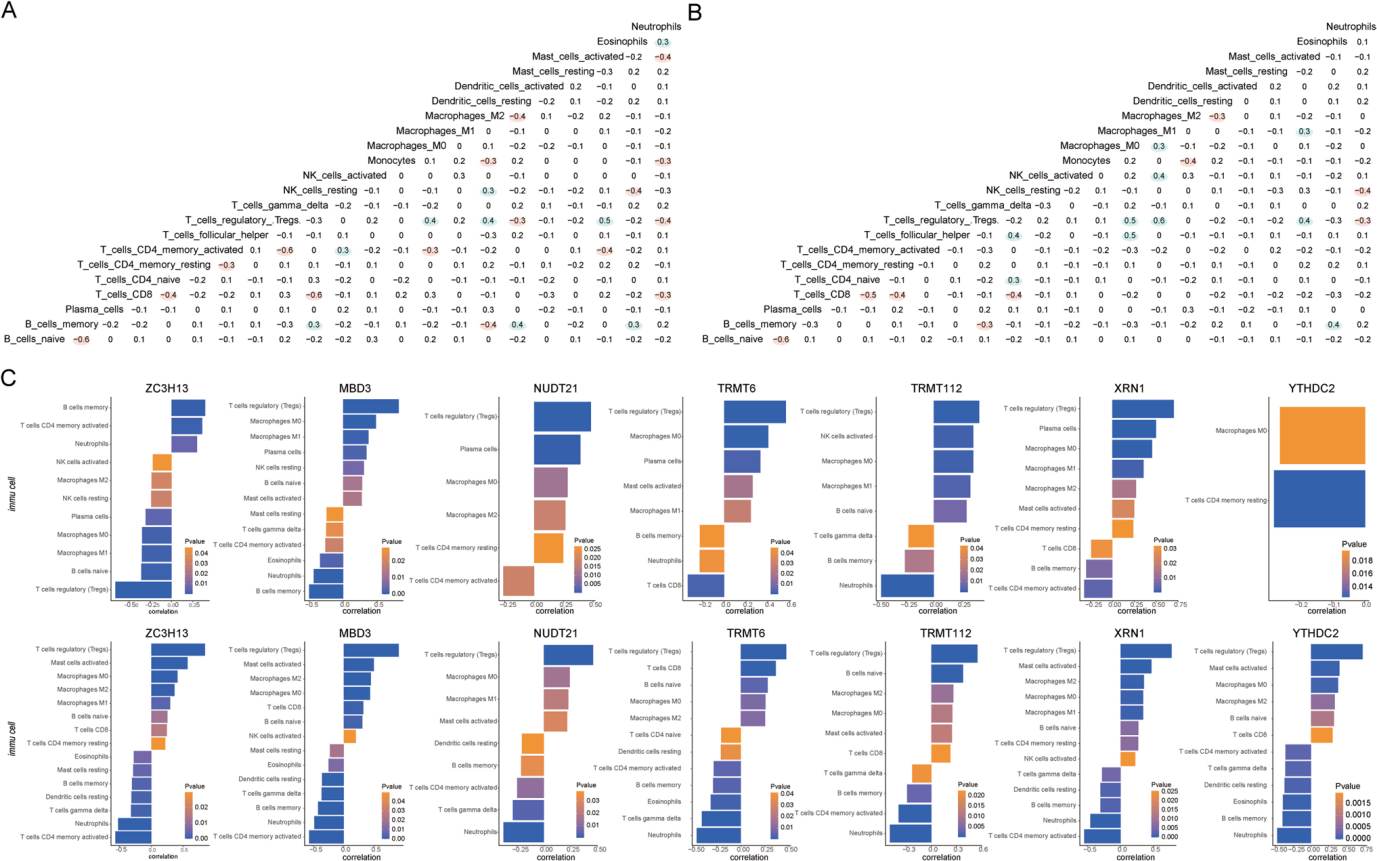
Immune signatures between the two RNA modification patterns CIBERSORT. **A** Bar graph of immune cell content between patients in the cluster 1 group and those in the cluster 2 group, with the cluster 2 samples in purple and the cluster 1 samples in blue. **B**, **C** Correlation of immune cell content between patients in cluster 1 group and patients in cluster 2 group; pink indicates positive correlation and blue indicates negative correlation. **D** Correlation between immune cells and characteristic genes in all MDD samples; node size represents significance and node color represents correlation. The horizontal axis is immune cells and the vertical axis is characteristic genes. **E**–**H** The first two terms of significant positive correlation and the first two terms of significant negative correlation of the correlation between characteristic genes and immune cell content in cluster 1 group patients, with immune cells on the horizontal axis and genes on the vertical axis. **I**–**L** The first two terms of significant positive correlation and negative correlation of the correlation between characteristic genes and immune cell content in cluster 1 group patients, with immune cells on the horizontal axis and genes on the vertical axis. MDD, major depressive disorder

Utilizing the CIBERSORT algorithm, it became discernibly clear that the associative dynamics of immune cellular constituents for individuals within the cluster1 assembly starkly deviated from those observed within the cluster 2 composition (*P* < 0.05, Fig. 13A, B). Simultaneously, an intricate evaluation was employed to dissect the relational intricacies between eight signature genes manifesting within the distinct RNA modification paradigms and the proportional makeup of immune entities. Astonishingly, the T cells, specifically the regulatory T cells (Tregs), evinced a potent association with the expression metrics of numerous signature genes. Furthermore, the relational fabric between these signature genes and immune constituents unveiled marked differences when juxtaposed across the two RNA modification spectra (*P* < 0.05, Fig. 13C).

**Fig. 14:**
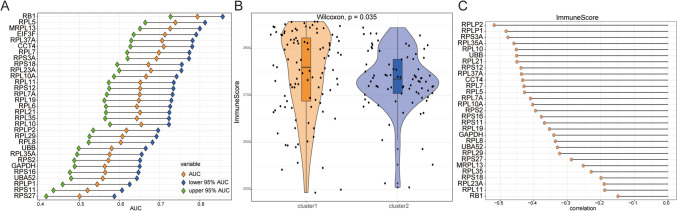
Calculation of the immune score. **A** AUC and 95 % AUC in the ROC curve of hub genes; green nodes are the lower 95 % AUC, blue is the upper 95 % AUC, orange is AUC, the horizontal axis is the AUC value, and the vertical axis is the lake hub gene. **B** Immune score of patients in cluster 1 group and cluster 2 group; the horizontal axis is grouping, the vertical axis is immune score, orange denotes cluster 1, and blue denotes cluster 2. **C** Correlation between immune score and hub gene in all MDD samples, horizontal axis is correlation; vertical axis is hub gene. MDD, major depressive disorder; ROC, receiver operating characteristic curve; AUC, area under the curve

In our quest to assess the discriminative potential of central genes across contrasting RNA modification paradigms, ROC curves were meticulously constructed for 31 such genes, followed by the computation of the AUC metrics. Remarkably, RPL37A, EIF3F, MRPL13, RPL5, RB1, among several other genes, adeptly differentiated between these two RNA modulatory archetypes (Fig. 14A). Concurrently, a discerning observation highlighted that the immunological indices of individuals within the cluster 1 configuration marginally surpassed those within the cluster 2 assemblage (*P* < 0.05, Fig. 14B). Delving further, an in-depth relational analysis was conducted between the expression magnitude of these central genes and the aforementioned immunological index. The outcome underscored a noticeable inverse association between RPL10, RPL35A, RPS3A, RPLP1, RPLP2, along with several other pivotal genes, and the immunological score (*P* < 0.05, Fig. 14C).

### Validation of Central Genes

**Fig. 15:**
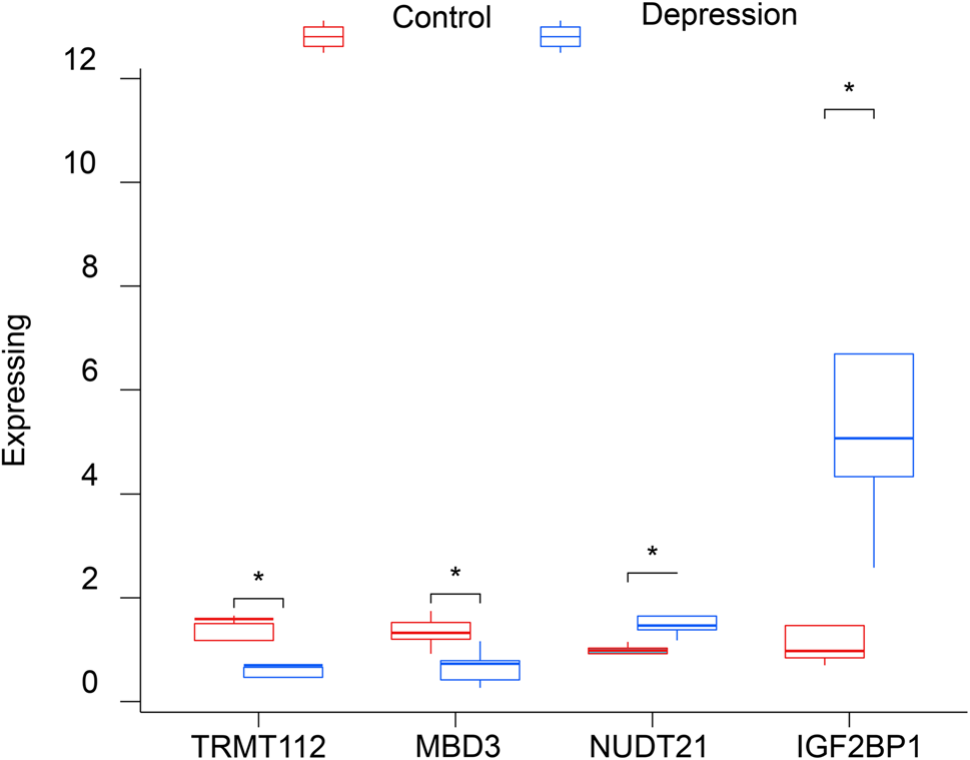
Quantitative real-time PCR analysis of the differences in expression of mRNA of the hub gene between controls and patients in MDD. Four genes were detected in the blood samples and the others were not detected. * *P* < 0.05. MDD, major depressive disorder

The transcriptional abundance of eight central genes was assessed in MDD and control blood specimens using quantitative PCR (qPCR) techniques. Among these genes, four distinct ones (TRMT112, MBD3, NUDT21, IGF2BP1) were identified, with NUDT21 and IGF2BP1 being notably significant, while the others were not detected. Among the two upregulated genes, TRMT112 and MBD3 exhibited statistical significance, and among the two downregulated genes, NUDT21 and IGF2BP1 displayed statistical significance (Fig. 15).

## Discussion

MDD, a widely observed psychiatric condition [1], is characterized by multifaceted symptoms such as mood perturbations, diminished pleasure or interest, as well as variations in physiological processes, cognitive functions, and psychomotor dynamics [2]. Contemporary research increasingly underscores the pivotal role of epigenetic modulation in MDD, with a specific emphasis on RNA-based epigenetic modifications. These RNA adjustments can influence nucleotide pairing, modulate RNA secondary conformation, and alter RNA’s propensity to interact with proteins [7]. Therefore, to construct a refined predictive model and identify potential prognostic indicators, it is imperative to comprehensively explore m6A/m5C/m1A-associated genes in the context of MDD.

In this study, we identified 29 differentially expressed RNA modification-associated genes (DERMGs) in MDD through bioinformatics analysis and unveiled two distinct RNA modification patterns based on signature genes for the first time. Subsequently, we constructed a risk signature comprising eight hub genes (ZC3H13, YTHDC2, TRMT112, MBD3, TRMT6, IGF2BP1, NUDT21, and XRN), of which four genes (TRMT112, MBD3, NUDT21, IGF2BP1) were detected in the blood samples, while the others were not detected. Additionally, we performed various analyses, including GO, KEGG, GSEA, and GSVA, to evaluate the enrichment associations of these genes with relevant pathways. Our findings indicated that these genes were enriched in processes such as reactive oxygen species biosynthesis, PI3K activity, NOD-like receptor signaling pathway, and mTORC1 signaling, confirming the role of RNA modification in MDD. Furthermore, we observed a significant positive correlation between IGF2BP1 and immune cells, specifically natural killer T cells, central memory CD4 T cells, and activated CD8 T cells, which has implications for depression immunotherapy.

Previous research has highlighted the significance of RNA modifications, including m6A, m5C, and m1A, in neurodegenerative disorders [39]. These modifications are introduced, removed, and recognized by specific proteins known as “writers,” “erasers,” and “readers.” For instance, m6A modifications involve writers such as METTL3/14, Wilms’ tumor 1-associating protein (WTAP), and ZC3H13, erasers like FTO and ALKBH5, and readers including YTHDC1/2, YTHDF1/2/3, and IGF2BP1/2/3. Similarly, RNA m1A modifications involve writers like TRMT61A/B, TRMT10C, and TRMT6, erasers such as ALKBH1/3/7 and FTO, and readers like YTHDF1/2/3 and YTHDC1 [40, 41]. Additionally, RNA m5C modifications are governed by NSUN enzymes, TET proteins, and YBX1 as writers, erasers, and readers, respectively [12]. These modifications have been implicated in synaptic plasticity, neural proliferation, cognitive processes, and stress responses within the brain [42].

To date, the demethylation of m6A facilitated by FTO in depressive conditions has garnered substantial academic scrutiny. An escalating volume of empirical works corroborate the instrumental functions of FTO across myriad biological paradigms [43, 44]. In our risk signature, among the eight hub genes, ZC3H13 and WTAP was the writer of m6A and IGF2BP, NUDT21, TRMT112, XRN, and YTHDC2 were m6A readers. They might play a key role in depression. First, as a writer of m6A, ZC3H13 together with WTAP is essential for assembling the ZC3H13-WTAP-VIRMA-HAKAI complex into the nucleus [44]. Moreover, previous studies showed that not only METTL3 [10] but also METTL14 [45] plays a crucial role in synaptic plasticity and stress-related disorders such as depressive behavior. Consequently, it stands to reason that ZC3H13, integral to our risk signature, could hold significant relevance in the context of MDD. However, the mechanism of ZC3H13 need to be further investigated in MDD. Furthermore, as the hub genes in our risk signature, NUDT21, TRMT112, IGF2BP, XRN, and YTHDC2 were m6A readers. Within this context, NUDT21, colloquially termed CFIm25, engages in the orchestration and subsequent production of circular RNAs (circRNAs), modulating both alternative splicing and the nuanced process of alternative polyadenylation (APA) [46]. Contemporary research posits that perturbations in the mechanisms driven by CFIm25, especially those concerning 3′-UTR-APA, might be implicated in neural anomalies and fibrotic manifestations [47]. After that, TRMT112 is a small evolutionarily conserved protein and current studies revealed that METTL5 and its partner TRMT112 are upregulated in various cancers [48, 49]. In addition, IGF2BP1 worked with c-MYC mRNA and E2F transcription factors, which was a transcriptional regulator adept at augmenting MIF transcription and played critical immunological roles, and then regulated T cells [50, 51]. Then, a recent bioinformatics analysis revealed similar evidence with our results, they found that elevated METTL16, YTHDC1, and YTHDC2 expression in prefrontal cortex of depressed patients and low IGF2BP1/2 expression in both normal and MDD patients [52]. And another study also, like our results, showed that the lack of association between XRN1 and YTHDC2 in the hypothalamus may contribute to metabolic disorders [53]. So we suppose that the m6A status of MDD is delicately balanced by the dynamic regulation among the m6A writer METTL3/5/14 and the partner TRMT112, eraser FTO/ALKBH5, and readers IGF2BP, XRN, YTHDC2, NUDT21. Besides that, as the hub genes in our risk, TRMT6 was m1A writer, which catalyzed m1A58 in tRNA together with TRMT61A [54]. Consequently, methyl-CpG-binding domain 3 proteins (MBD3) serve as interpreters for m5C, offering foundational and DNA-affinity characteristics to the nucleosome reorganization and deacetylation (NuRD) assembly, associating with CpG domains [55]. Therefore, the central genes within our hazard assessment could be instrumental in the molecular dynamics of MDD.

Furthermore, to analyze the effects of DEGs between patients with two RNA modification patterns on the biological-related functions of MDD patients, we first constructed a PPI related to differentially expressed genes. The PPI network included 1597 interaction pairs and 507 differentially expressed genes. Functioning as an transcriptional repressor, retinoblastoma 1(RB1), it modulates the transcription of cell-cycle-related genes via its synergy with the E2F transcription factor lineage, inhibiting transcription amid differentiation and stress situations [56]. And UBA52 (ubiquitin-60S ribosomal protein L40, RPL40) encodes one UB unit fused to a ribosomal protein, which plays a role in enhancing ribosome biogenesis and mitochondrial homeostasis-induced cell death [57]. Then, we first constructed a risk signature related to differentially expressed genes. The findings revealed that RPL37A, EIF3F, MRPL13, RPL5, and RB1, along with other genes, adeptly differentiated between two distinct RNA modification templates. Concurrently, evidence indicated a negative association between RPL10, RPL35A, RPS3A, RPLP1, RPLP2 and several pivotal genes with the immune metric (*P* < 0.05). Positioned as a central gene in this risk profile, RB1 orchestrates cell-cycle gene expression by interfacing with the E2F transcription factor lineage [56]. In addition, EIF3 plays an oncogenic role through the regulation of PI3K/Akt/NF-κB signaling. It is also consistent with our results about GO and KEGG analysis in hub gene. Furthermore, RPL10, RPL35A, RPS3A, RPLP1, and RPLP2 belong to the ribosomal protein (RP) family, promoted tumorigenesis and aging in brain among them [58], and RPL5 functions as a monitor for ribosomal disruptions and potentially influences E2F transcription factor 1 (E2F1) [59]. Our data corroborates the notion that it holds a pivotal function in MDD. So our study establishes a model that reveals how an m6A/m1A/m5C-modified RNA epigenetic translational though two RNA modification patterns on the biological related functions of MDD.

Research has demonstrated that the PI3K, NOD, and mTORC1 signaling pathways are involved in the development of depression. Our experiments, utilizing functional enrichment analysis, have revealed that key genes are also primarily concentrated in the PI3K, NOD, and mTORC1 signaling pathways. In prior research, both YTHDF2 and FTO were observed to enhance the self-renewal and proliferation capacities of NSCs via modulation of the PI3K/AKT and JAK/STAT signaling cascades [60]. Moreover, mTORC1 augments the stabilization of the MTC that encompasses METTL3, METTL14, WTAP, and RMB15/RBM15B [56]. Additionally, within the brain, the PI3K/Akt/mTOR signaling cascade holds significant importance in the etiology and treatment of MDD [61]. Moreover, some studies reported that METTL14 represses colorectal cancer (CRC) development via the PI3K/Akt signaling, upregulated by brain-derived neurotrophic factor (BDNF), which revealed similar evidence with our GO results. Subsequently, BDNF intricately orchestrates not merely the MAPK/ERK and PI3K/Akt cascades but also stimulates the mTOR pathway [60, 61]. Evidently, the HPA axis critically oversees oxidative stress (OS), with its regulation steered by GR, dictating epigenetic inscription and structuring in MDD [7]. Our findings corroborate the pivotal influence of m6A modification within the context of depression.

Our research may contribute to depression immunotherapy. At present, immunotherapy approaches have yielded great success in MDD, but the outcomes for the majority of patients remain unsatisfactory. In our results, immune infiltration and correlation analysis between experimental and control group revealed the most significantly positively correlated were IGF2BP1 and natural killer T cells, central memory CD4 T cells, and activated CD8 T cell (*P* < 0.0.5). Moreover, the immune integration and interrelation assessment, focusing on dual RNA modification patterns in MDD sufferers, indicated a pronounced correlation of YTHDC2 with the majority of immune cells for individuals in the cluster 1 category. Conversely, the association of YTHDC2 with immune cells in those belonging to the cluster 2 group appeared to be insubstantial (*P* < 0.05, Fig. 12D, E). Concurrently, the interdependence between pivotal genes and Tregs was distinctly associated with the expression metrics of several distinguishing genes. And current studies also like our results showed that T cell have been indicated to be related to depression and mainly through epigenetic control [62]. During penetration, brain-native T cells exhibit linkage with autoantigens present in the cerebral domain and are preconditioned to express anti-inflammatory agents along with neural growth elements [62, 63]. And another study indicated that T cell was closely related to the IGF2BP1 [64] which is same with our result that significant positive correlation between IGF2BP1 and T cell. It is consistent with our results. Consequently, our research formulates a paradigm elucidating the orchestration of m6A/m1A/m5C-modified RNA epigenetic translational regulators in modulating overall translation within expanding T cells. Additionally, our findings potentially illuminate an uncharted therapeutic nexus, potentially mitigating a myriad of T cell-associated inflammatory conditions, introducing a novel tactic for MDD immunotherapeutic interventions.

Consequently, a nomographic representation encompassing eight pivotal genes was devised, identifying four within the context of MDD. Simultaneously, AUC values were assessed, and ROC trajectories for the 31 central genes were delineated to appraise their potential in differentiating disparate RNA modification profiles. The data suggest that the projected risk quotient manifests noteworthy predictive efficacy. The prognostic line consistently surpassed the lavender threshold, alluding to the potential clinical advantages of utilizing this nomographic model for MDD patients. However, the roles of other m6A-associated proteins remain under-explored and warrant deeper scrutiny within the MDD spectrum.

In the context of MDD, m6A modifications have garnered substantial attention, especially the role of FTO in m6A demethylation. Our risk signature includes ZC3H13 and WTAP as writers of m6A, and IGF2BP1, NUDT21, TRMT112, XRN, and YTHDC2 as m6A readers. These genes may play crucial roles in depression, but further investigation is needed to fully understand their mechanisms. Additionally, our study highlights the potential therapeutic implications of targeting these RNA modifications in MDD.

A protein–protein interaction (PPI) network of differentially expressed genes was also constructed, identifying central genes such as RB1 and UBA52. Subsequently, a risk signature related to these central genes was devised, with genes like RPL37A, EIF3F, MRPL13, RPL5, and RB1 showing the ability to differentiate between distinct RNA modification patterns. Additionally, a negative association between some of these central genes and an immunological score was observed, indicating their potential relevance in the context of MDD.

In conclusion, our study sheds light on the role of RNA modifications in MDD and provides insights into potential therapeutic targets and prognostic indicators. The complex interplay between RNA modification-associated genes and immune cells further underscores the potential of immunotherapy in MDD. However, further research is needed to fully elucidate the roles of these genes and their implications for MDD treatment.

## Limitations

Several limitations are present in this study. Firstly, due to the high costs of sequencing and the relatively small sample size, the results may lack sufficient representativeness. Secondly, since the study primarily relied on bioinformatics analysis, the findings remain largely theoretical, and their accuracy requires confirmation through experiments. Thirdly, owing to the lack of adequate clinical data, only a small sample consisting of six depression patients and six normal individuals was analyzed, and the specific roles of genes related to m6A/m5C/m1A in MDD have not been conclusively determined. Therefore, further studies with larger sample sizes are necessary.

## Conclusions

In summary, a nomogram model was developed, incorporating eight hub genes, and two distinct RNA modification patterns were distinguished. These patterns may have been regulated by various factors, including m6A writers (METTL3/5/14) and their partner (TRMT112), erasers (FTO/ALKBH5), and readers (IGF2BP, XRN, YTHDC2, NUDT21). However, the mechanisms underlying RNA modification in MDD are still relatively unexplored and warrant further investigation.

## Supplementary Information

Below is the link to the electronic supplementary material.

Supplementary file1 (DOCX 13 KB)

## Data Availability

The datasets presented in this study can be found in the online repositories. The names of the repository/repositories and accession numbers (s) can be found in the article and supplementary material.

## References

[1] Herrman H et al (2022) Time for united action on depression: a Lancet-World Psychiatric Association Commission. The Lancet 399(10328):957–1022 10.1016/S0140-6736(21)02141-3 35180424

[2] Holtzheimer PE, Mayberg HS (2011) Stuck in a rut: rethinking depression and its treatment. Trends Neurosci 34(1):1–9 21067824 10.1016/j.tins.2010.10.004 PMC3014414

[3] Nestler EJ et al (2002) Neurobiology of depression. Neuron 34(1):13–25 11931738 10.1016/s0896-6273(02)00653-0

[4] Taby R, Issa JPJ (2010) Cancer epigenetics. CA: a cancer journal for clinicians 60(6):376–392 20959400 10.3322/caac.20085

[5] Chan RF et al (2020) Cell type–specific methylome-wide association studies implicate neurotrophin and innate immune signaling in major depressive disorder. Biol Psychiat 87(5):431–442 31889537 10.1016/j.biopsych.2019.10.014 PMC9933050

[6] Smeeth D et al (2021) The role of epigenetics in psychological resilience. Lancet Psychiatry 8(7):620–629 33915083 10.1016/S2215-0366(20)30515-0 PMC9561637

[7] Gagnidze K et al (2018) A new chapter in genetic medicine: RNA editing and its role in disease pathogenesis. Trends Mol Med 24(3):294–303 29483039 10.1016/j.molmed.2018.01.002

[8] Roundtree IA et al (2017) Dynamic RNA modifications in gene expression regulation. Cell 169(7):1187–1200 28622506 10.1016/j.cell.2017.05.045 PMC5657247

[9] He C (2010) Grand challenge commentary: RNA epigenetics? Nat Chem Biol 6(12):863–865 21079590 10.1038/nchembio.482

[10] Engel M et al (2018) The role of m6A/m-RNA methylation in stress response regulation. Neuron 99(2):389–403 e9 30048615 10.1016/j.neuron.2018.07.009 PMC6069762

[11] Vissers C et al (2020) The epitranscriptome in stem cell biology and neural development. Neurobiol Dis 146:105139 33065280 10.1016/j.nbd.2020.105139 PMC7686257

[12] Blaze J et al (2021) Neuronal Nsun2 deficiency produces tRNA epitranscriptomic alterations and proteomic shifts impacting synaptic signaling and behavior. Nat Commun 12(1):4913 34389722 10.1038/s41467-021-24969-x PMC8363735

[13] Yi Z et al (2012) Blood-based gene expression profiles models for classification of subsyndromal symptomatic depression and major depressive disorder. PLoS One 7(2):e31283 22348066 10.1371/journal.pone.0031283 PMC3278427

[14] Leday GG et al (2018) Replicable and coupled changes in innate and adaptive immune gene expression in two case-control studies of blood microarrays in major depressive disorder. Biol Psychiat 83(1):70–80 28688579 10.1016/j.biopsych.2017.01.021 PMC5720346

[15] Spijker S et al (2010) Stimulated gene expression profiles as a blood marker of major depressive disorder. Biol Psychiat 68(2):179–186 20471630 10.1016/j.biopsych.2010.03.017

[16] Zhang D et al (2022) Peripheral blood circular RNAs as a biomarker for major depressive disorder and prediction of possible pathways. Front Neurosci 16:844422 35431783 10.3389/fnins.2022.844422 PMC9009243

[17] Leek JT et al (2012) The sva package for removing batch effects and other unwanted variation in high-throughput experiments. Bioinformatics 28(6):882–883 22257669 10.1093/bioinformatics/bts034 PMC3307112

[18] Zhao L-Y et al (2020) Mapping the epigenetic modifications of DNA and RNA. Protein Cell 11(11):792–808 32440736 10.1007/s13238-020-00733-7 PMC7647981

[19] Ritchie ME et al (2015) limma powers differential expression analyses for RNA-sequencing and microarray studies. Nucleic Acids Res 43(7):e47–e47 25605792 10.1093/nar/gkv007 PMC4402510

[20] Ashburner M et al (2000) Gene ontology: tool for the unification of biology. Nat Genet 25(1):25–29 10802651 10.1038/75556 PMC3037419

[21] Kanehisa M, Goto S (2000) KEGG: Kyoto encyclopedia of genes and genomes. Nucleic Acids Res 28(1):27–30 10592173 10.1093/nar/28.1.27 PMC102409

[22] Yu G et al (2012) ClusterProfiler: an R package for comparing biological themes among gene clusters. Omics: a journal of integrative biology 16(5):284–287 22455463 10.1089/omi.2011.0118 PMC3339379

[23] Wu T et al (2021) clusterProfiler 4.0: A universal enrichment tool for interpreting omics data*.* The innovation 2(3): 100141 10.1016/j.xinn.2021.100141 PMC8454663 34557778

[24] Barbie DA et al (2009) Systematic RNA interference reveals that oncogenic KRAS-driven cancers require TBK1. Nature 462(7269):108–112 19847166 10.1038/nature08460 PMC2783335

[25] Newman AM et al (2019) Determining cell type abundance and expression from bulk tissues with digital cytometry. Nat Biotechnol 37(7):773–782 31061481 10.1038/s41587-019-0114-2 PMC6610714

[26] Yoshihara K et al (2013) Inferring tumour purity and stromal and immune cell admixture from expression data. Nat Commun 4:2612 24113773 10.1038/ncomms3612 PMC3826632

[27] Wei T et al (2017) Package ‘corrplot.’ Statistician 56(316):e24

[28] Milošević D et al (2022) The application of uniform manifold approximation and projection (UMAP) for unconstrained ordination and classification of biological indicators in aquatic ecology. Sci Total Environ 815:152365 34963591 10.1016/j.scitotenv.2021.152365

[29] Subramanian A et al (2005) Gene set enrichment analysis: a knowledge-based approach for interpreting genome-wide expression profiles. Proc Natl Acad Sci 102(43):15545–15550 16199517 10.1073/pnas.0506580102 PMC1239896

[30] Liberzon A et al (2015) The molecular signatures database hallmark gene set collection. Cell Syst 1(6):417–425 26771021 10.1016/j.cels.2015.12.004 PMC4707969

[31] Hänzelmann S, Castelo R, Guinney J (2013) GSVA: gene set variation analysis for microarray and RNA-seq data. BMC Bioinform 14:1–15 10.1186/1471-2105-14-7 PMC3618321 23323831

[32] Mering CV et al (2003) STRING: a database of predicted functional associations between proteins. Nucleic Acids Res 31(1):258–261 12519996 10.1093/nar/gkg034 PMC165481

[33] Shannon P et al (2003) Cytoscape: a software environment for integrated models of biomolecular interaction networks. Genome Res 13(11):2498–2504 14597658 10.1101/gr.1239303 PMC403769

[34] Chin C-H et al (2014) cytoHubba: identifying hub objects and sub-networks from complex interactome. BMC Syst Biol 8(4):1–7 25521941 10.1186/1752-0509-8-S4-S11 PMC4290687

[35] Lu TX, Rothenberg ME (2018) MicroRNA. J Allergy Clin Immunol 141(4):1202–1207 29074454 10.1016/j.jaci.2017.08.034 PMC5889965

[36] Robin X et al (2011) pROC: an open-source package for R and S+ to analyze and compare ROC curves. BMC Bioinform 12:1–8 10.1186/1471-2105-12-77 PMC3068975 21414208

[37] Zhang H, Meltzer P, Davis S (2013) RCircos: an R package for Circos 2D track plots. BMC Bioinform 14:1–5 10.1186/1471-2105-14-244 PMC3765848 23937229

[38] Yu G (2020) Gene ontology semantic similarity analysis using GOSemSim. Stem Cell Transcriptional Netw: Methods and Protoc 207–215 10.1007/978-1-0716-0301-7_11 31960380

[39] Shafik AM, Allen EG, Jin P (2022) Epitranscriptomic dynamics in brain development and disease. Mol Psychiatry 27(9):3633–3646 35474104 10.1038/s41380-022-01570-2 PMC9596619

[40] Deng X et al (2023) The roles and implications of RNA m6A modification in cancer. Nat Rev Clin Oncol 1–20 10.1038/s41571-023-00774-x PMC12466201 37221357

[41] Li J, Zhang H, Wang H (2022) N1-methyladenosine modification in cancer biology: current status and future perspectives. Comput Struct Biotechnol J 20:6578-6585 10.1016/j.csbj.2022.11.045 PMC9712505 36467585

[42] Chokkalla AK et al (2019) Transient focal ischemia significantly alters the m6A epitranscriptomic tagging of RNAs in the brain. Stroke 50(10):2912–2921 31436138 10.1161/STROKEAHA.119.026433 PMC6759411

[43] Liu S et al (2021) Fat mass and obesity-associated protein regulates RNA methylation associated with depression-like behavior in mice. Nat Commun 12(1):6937 34836959 10.1038/s41467-021-27044-7 PMC8626436

[44] Chelmicki T et al (2021) m6A RNA methylation regulates the fate of endogenous retroviruses. Nature 591(7849):312–316 33442060 10.1038/s41586-020-03135-1

[45] Koranda JL et al (2018) Mettl14 is essential for epitranscriptomic regulation of striatal function and learning. Neuron 99(2):283–292 (e5) 30056831 10.1016/j.neuron.2018.06.007 PMC6082022

[46] Brumbaugh J et al (2018) Nudt21 controls cell fate by connecting alternative polyadenylation to chromatin signaling. Cell 172(1):106–120 (e21) 29249356 10.1016/j.cell.2017.11.023 PMC5766360

[47] Masamha CP (2023) The emerging roles of CFIm25 (NUDT21/CPSF5) in human biology and disease. Wiley Interdisc Rev: RNA 14(3):e1757 10.1002/wrna.1757 PMC9925614 35965101

[48] Van Tran N et al (2019) The human 18S rRNA m6A methyltransferase METTL5 is stabilized by TRMT112. Nucleic Acids Res 47(15):7719–7733 31328227 10.1093/nar/gkz619 PMC6735865

[49] Peng H et al (2022) N 6-methyladenosine (m6A) in 18S rRNA promotes fatty acid metabolism and oncogenic transformation. Nat Metab 4(8):1041–1054 35999469 10.1038/s42255-022-00622-9

[50] Mao Y et al (2023) Inhibition of IGF2BP1 attenuates renal injury and inflammation by alleviating m6A modifications and E2F1/MIF pathway. Int J Biol Sci 19(2):593 36632449 10.7150/ijbs.78348 PMC9830505

[51] Müller S et al (2020) The oncofetal RNA-binding protein IGF2BP1 is a druggable, post-transcriptional super-enhancer of E2F-driven gene expression in cancer. Nucleic Acids Res 48(15):8576–8590 32761127 10.1093/nar/gkaa653 PMC7470957

[52] Lv J et al (2023) Role of N6-methyladenosine modification in central nervous system diseases and related therapeutic agents. Biomed Pharmacother 162:114583 36989722 10.1016/j.biopha.2023.114583

[53] Takaoka S et al (2021) Neuronal XRN1 is required for maintenance of whole-body metabolic homeostasis. iScience 24:103151 10.1016/j.isci.2021.103151 PMC8496175 34646989

[54] Li X, Xiong X, Yi C (2017) Epitranscriptome sequencing technologies: decoding RNA modifications. Nat Methods 14(1):23–31 10.1038/nmeth.4110 28032622

[55] Leighton GO et al (2022) Densely methylated DNA traps methyl-CpG-binding domain protein 2 but permits free diffusion by methyl-CpG-binding domain protein 3. J Biol Chem 298(10):102428 10.1016/j.jbc.2022.102428 PMC9520026 36037972

[56] Ishak CA et al (2016) An RB-EZH2 complex mediates silencing of repetitive DNA sequences. Mol Cell 64(6):1074–1087 27889452 10.1016/j.molcel.2016.10.021 PMC5340194

[57] Tiwari S et al (2023) UBA52 attunes VDAC1-mediated mitochondrial dysfunction and dopaminergic neuronal death. ACS Chem Neurosci 14(5):839–850 36755387 10.1021/acschemneuro.2c00579

[58] Suzuki M et al (2022) Upregulation of ribosome complexes at the blood-brain barrier in Alzheimer’s disease patients. J Cereb Blood Flow Metab 42(11):2134–2150 35766008 10.1177/0271678X221111602 PMC9580172

[59] Ma X, Li Y, Zhao B (2022) Ribosomal protein L5 (RPL5)/E2F transcription factor 1 (E2F1) signaling suppresses breast cancer progression via regulating endoplasmic reticulum stress and autophagy. Bioengineered 13(4):8076–8086 35293275 10.1080/21655979.2022.2052672 PMC9161874

[60] Fries GR et al (2023) Molecular pathways of major depressive disorder converge on the synapse. Mol Psychiatry 28(1):284–297 36203007 10.1038/s41380-022-01806-1 PMC9540059

[61] Jiang X et al (2021) The role of m6A modification in the biological functions and diseases. Signal Transduct Target Ther 6(1):74 33611339 10.1038/s41392-020-00450-x PMC7897327

[62] Liu Y et al (2022) tRNA-m1A modification promotes T cell expansion via efficient MYC protein synthesis. Nat Immunol 23(10):1433–1444 36138184 10.1038/s41590-022-01301-3

[63] Salvetat N et al (2021) Phosphodiesterase 8A to discriminate in blood samples depressed patients and suicide attempters from healthy controls based on A-to-I RNA editing modifications. Transl Psychiatry 11(1):255 33931591 10.1038/s41398-021-01377-9 PMC8087806

[64] Liu Y et al (2022) Allosteric regulation of IGF2BP1 as a novel strategy for the activation of tumor immune microenvironment. ACS Cent Sci 8(8):1102–1115 36032766 10.1021/acscentsci.2c00107 PMC9413439

