# Supplementary material for: m6A/m1A/m5C-Associated Methylation Alterations and Immune Profile in MDD
Source: Mol Neurobiol. 2024 Mar 8;61(10):8000–25. doi: 10.1007/s12035-024-04042-6 (PMC11415454; doi:10.1007/s12035-024-04042-6)
Supplement: Supplementary file 1 — Supplementary file1 (DOCX 13 KB) [file 12035_2024_4042_MOESM1_ESM.docx]

Table S1．m1A, m5C, and m6A related genes

| Gene | Type | Modifications | |
| --- | --- | --- | --- |
| TRMT6 | writer | | m1A |
| TRMT61A | writer | | m1A |
| TRMT61B | writer | | m1A |
| TRMT10C | writer | | m1A |
| ALKBH1 | eraser | | m1A |
| ALKBH3 | eraser | | m1A |
| NSUN1 | writer | | m5C |
| NSUN2 | writer | | m5C |
| NSUN3 | writer | | m5C |
| NSUN4 | writer | | m5C |
| NSUN5 | writer | | m5C |
| NSUN6 | writer | | m5C |
| NSUN7 | writer | | m5C |
| DNMT1 | writer | | m5C |
| DNMT2 | writer | | m5C |
| DNMT3A | writer | | m5C |
| DNMT3B | writer | | m5C |
| ALYREF | reader | | m5C |
| YBX1 | reader | | m5C |
| MBD1 | reader | | m5C |
| MBD2 | reader | | m5C |
| MBD3 | reader | | m5C |
| MBD4 | reader | | m5C |
| MECP2 | reader | | m5C |
| NEIL1 | reader | | m5C |
| NTHL1 | reader | | m5C |
| SMUG1 | reader | | m5C |
| TDG | reader | | m5C |
| UHRF1 | reader | | m5C |
| UHRF2 | reader | | m5C |
| UNG | reader | | m5C |
| ZBTB33 | reader | | m5C |
| ZBTB4 | reader | | m5C |
| TET1 | eraser | | m5C |
| TET2 | eraser | | m5C |
| TET3 | eraser | | m5C |
| METTL3 | writer | | m6A |
| METTL14 | writer | | m6A |
| WTAP | writer | | m6A |
| VIRMA | writer | | m6A |
| ZC3H13 | writer | | m6A |
| CBLL1 | writer | | m6A |
| RBM15 | writer | | m6A |
| RBM15B | writer | | m6A |
| METTL16 | writer | | m6A |
| ZCCHC4 | writer | | m6A |
| PCIF1 | writer | | m6A |
| FTO | eraser | | m6A |
| ALKBH5 | eraser | | m6A |
| ALKBH3 | eraser | | m6A |
| YTHDF1 | reader | | m6A |
| YTHDF2 | reader | | m6A |
| YTHDF3 | reader | | m6A |
| YTHDC1 | reader | | m6A |
| YTHDC2 | reader | | m6A |
| YTHDC3 | reader | | m6A |
| HNRNPA2B1 | reader | | m6A |
| HNRNPC | reader | | m6A |
| RBMX | reader | | m6A |
| IGF2BP1 | reader | | m6A |
| IGF2BP2 | reader | | m6A |
| IGF2BP3 | reader | | m6A |
| FMR1 | reader | | m6A |
| PRRC2A | reader | | m6A |
| EIF3A | reader | | m6A |
| EIF3B | reader | | m6A |
| EIF3H | reader | | m6A |
| LRPPRC | reader | | m6A |
| SRSF3 | reader | | m6A |
| NXF1 | reader | | m6A |
| TRMT112 | reader | | m6A |
| NUDT21 | reader | | m6A |
| CPSF6 | reader | | m6A |
| SETD2 | reader | | m6A |
| SRSF10 | reader | | m6A |
| XRN1 | reader | | m6A |
